# Ultrafast Synthesis of Metal-Layered Hydroxides in a Dozen Seconds for High-Performance Aqueous Zn (Micro-) Battery

**DOI:** 10.1007/s40820-022-01004-2

**Published:** 2023-01-09

**Authors:** Xiangyang Li, Fangshuai Chen, Bo Zhao, Shaohua Zhang, Xiaoyu Zheng, Ying Wang, Xuting Jin, Chunlong Dai, Jiaqi Wang, Jing Xie, Zhipan Zhang, Yang Zhao

**Affiliations:** Key Laboratory of Cluster Science Ministry of Education of China, Beijing Key Laboratory of Photoelectronic/Electrophotonic Conversion Materials, School of Chemistry and Chemical Engineering, Beijing Institute of Technology, Beijing, 100081 People’s Republic of China

**Keywords:** Ultrafast synthesis, Thermal shock, Metal-layered hydroxides, Zn (micro-) battery

## Abstract

Efficient synthesis of transition metal hydroxides on conductive substrate is essential for enhancing their merits in industrialization of energy storage field. However, most of the synthetic routes at present mainly rely on traditional bottom-up method, which involves tedious steps, time-consuming treatments, or additional alkaline media, and is unfavorable for high-efficiency production. Herein, we present a facile, ultrafast and general avenue to synthesize transition metal hydroxides on carbon substrate within 13 s by Joule-heating method. With high reaction kinetics caused by the instantaneous high temperature, seven kinds of transition metal-layered hydroxides (TM-LDHs) are formed on carbon cloth. Therein, the fastest synthesis rate reaches ~ 0.46 cm^2^ s^−1^. Density functional theory calculations further demonstrate the nucleation energy barriers and potential mechanism for the formation of metal-based hydroxides on carbon substrates. This efficient approach avoids the use of extra agents, multiple steps, and long production time and endows the LDHs@carbon cloth with outstanding flexibility and machinability, showing practical advantages in both common and micro-zinc ion-based energy storage devices. To prove its utility, as a cathode in rechargeable aqueous alkaline Zn (micro-) battery, the NiCo LDH@carbon cloth exhibits a high energy density, superior to most transition metal LDH materials reported so far.

**Supplementary Information:**

The online version contains supplementary material available at 10.1007/s40820-022-01004-2.

## Highlights

- An efficient and general strategy for ultrafast synthesis of seven transition metal layered hydroxides on conductive substrates is proposed, which avoids the use of additional reagents, multiple steps and long production time in the traditional bottom-up method.
- The whole synthesis process only takes as fast as approximately 13 s, and its synthesis rate reaches ~ 0.46 cm^2^ s^−1^.
- The NiCo LDH@CC as a demonstration example is selected as cathode material for aqueous alkaline Zn (micro-) battery, which exhibits an outstanding performance.

**Supplementary Information:**

The online version contains supplementary material available at 10.1007/s40820-022-01004-2.

## Graphical abstract

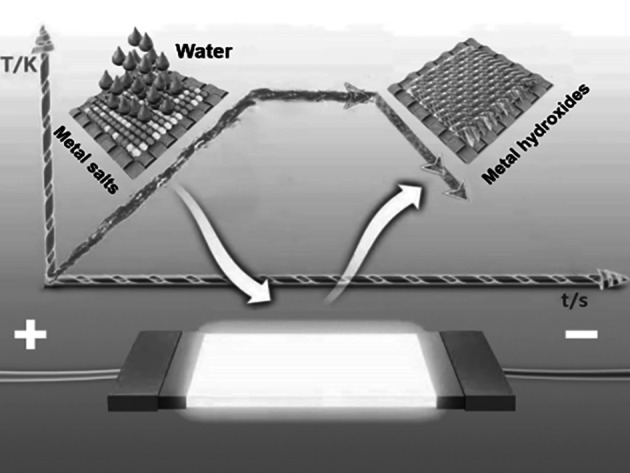

## Introduction

Nowadays, advanced renewable energy storage systems have attracted great attentions due to the depletion of fossil energy and environmental problems. Correspondingly, various advanced energy storage devices, such as metal ions (lithium [1], sodium [2], potassium [3], zinc [4–6], magnesium [7], calcium [8]) batteries, supercapacitors [9–11] and metal-air batteries [12], have been developed rapidly and innovatively. In these energy storage devices, the electrodes play a key role in electrochemical performance. It is well known that one of the most efficient ways to improve performance of electrodes is to design and develop new electrode materials with special micro-/nanostructures, including zero-dimensional (0D) quantum dots [13], one-dimensional (1D) nanotubes [14], nanowires [15] and nanorods [16], two-dimensional (2D) nanosheets [17–19], etc. Among them, 2D nanosheets with short ion diffusion pathways and abundant accessible active sites are attracting more and more attentions [20]. Due to their high redox activity, easily customize components and low-cost properties, transition metal-layered hydroxides (TM-LDHs) nanosheets have been proven to be promising electrochemically active materials [21–23]. Unfortunately, the low intrinsic conductivity of TM-LDHs inhibits the fast electron transfer in the electrodes, leading to poor cycle stability. To address this issue, a common and effective approach is to *in situ* grow TM-LDHs on the conductive substrates (e.g., carbon cloth [24], carbon nanotubes [25] and Ni foam [26]), which can not only form 3D conductive network to enhance the transfer of electrons, but also make the contact between the electrode and electrolyte more effective, thereby exhibiting great potential application in energy storage devices.

To date, one of the most common fabrication approaches for TM-LDHs on the conductive substrate is bottom-up method, including hydrothermal synthesis [27–30], co-precipitation [31] and electrochemical deposition [32]. Specifically, the hydrothermal process is widely considered as a simple and facile method to obtain the high-purity TM-LDHs nanosheets on conductive substrate. However, it usually involves hours of reaction due to the kinetic limitation and introduction of precipitators (e.g., urea, ammonia, hexamethylenetetramine and methanol) to provide hydroxyl anions [33]. The hydrothermal reaction requires high temperature and high pressure, which is too harsh and dangerous to industrial manufacturing. In recent years, co-precipitation synthesis and electrochemical deposition synthesis have been developed to be the fast and efficient synthesis routes to prepare high-quality TM-LDHs nanosheets. In co-precipitation synthesis, the target phase of LDHs can in situ grow on carbon substrate by introducing co-precipitation agents (e.g., NaOH, NH_3_‧H_2_O or Na_2_CO_3_) and carbon substrate into metal salt solution. Unfortunately, the deposition selectivity of the target phase is poor as well as it is prone to agglomeration, which usually requires a tedious post-processing process, such as thermal treatment and aging process, to uniformly deposit on the conductive substrate. Moreover, the introduction of coprecipitating agents may be harmful to environment and also increases the cost of scale-up production. By contrast, the electrochemical deposition method has been proven to be a time-saving (several minutes), economical and facile synthesis method to fabricate TM-LDHs nanosheets. Nevertheless, it still suffers from inhomogeneous plating due to inevitable concentration polarization, and poor crystallinity of the target phase caused by uncontrolled nucleation growth. To sum up, it is of great significance for industrial production to explore and develop a simple, time-saving, and efficient large-scale method to achieve high-quality and well-structured TM-LDHs on conductive substrate. In the past few years, Chen et al. reported a series of works for rapid deposition of nanoparticles on substrates by simple Joule heating, which provided a new method for ultrafast nanofabrication [34–38]. However, this method applied was carried out in air medium or inert atmosphere; it is essential to extend this method to other systems for efficient synthesis of new materials.

Herein, we report an ultrafast and highly efficient avenue to in situ synthesize 2D TM-LDHs on treated conductive carbon cloth (named as TM-LDH@CC) via Joule-heating method in the metal salt solution. A hydrophilic carbon cloth pretreated by instant thermal shock is used as substrate and immersed in transition metal salt solution. When the high electronic current is applied across the carbon substrate, abundant Joule heat can be generated around the carbon cloth to drive the hydrolysis of metal salt, leading to rapid formation and growth of the target phase on substrate. The whole synthesis process only takes as fast as approximately 13 s, and its synthesis rate reaches ~ 0.46 cm^2^ s^−1^, which is more efficient than other conventional methods reported previously. Density functional theory (DFT) calculations demonstrate that the heating energy generated around the carbon cloth is far higher than the nucleation energy barriers of metal-based layered hydroxides phase. To prove utility, the NiCo LDH@CC as the demonstration example is selected as cathode material for aqueous alkaline zinc ion battery, which exhibits high energy density of 301.7 Wh kg^−1^, prominent long-term stability (81.4% retention after 5,000 cycles at 15 A g^−1^), and outperforms most of the previous works for aqueous zinc ion battery. Moreover, based on superior mechanical stability and processability, the in-plane flexible quasi-solid-state zinc ion micro-battery can be easily assembled by using NiCo LDH@CC as cathode material, Zn foil as anode and PVA-KOH as electrolyte. The micro-devices exhibit a considerable capacity of 92 µAh cm^−2^ with high energy density of 146 µWh cm^−2^ and excellent cycling performance (91.2% capacity retention after 300 cycles). This study may open a new avenue for ultrafast and low-cost synthesis of metal-based layered hydroxide-based composites.

## Experimental Section

### Materials

Nickel(II) chloride hexahydrate, cobalt (II) chloride hexahydrate, ferrous (III) chloride tetrahydrate, anhydrous manganese (II) chloride, PVA and KOH were purchased from Aladdin (Shanghai, China). After Joule-heating treatment for 2 s, carbon clothes (CC) and graphite paper were cut to 7.5 × 0.8 cm^2^.

### Synthesis of Metal LDH@Carbon Substrate Composites

At room temperature, 2.5 mM nickel (II) chloride hexahydrate and 2.5 mM cobalt (II) chloride hexahydrate were mixed in deionized water under magnetic stirring to prepare 0.25 mol L^−1^ NiCl_2_/CoCl_2_. Next, 10 g of the mixture was transferred into a ceramic boat. A piece of treated CC was dipped into the mixed metal salt solutions for 2 min. Then, the Joule-heating process was achieved by directly applying power (~ 300 W) to the both ends of treated CC for about 13 s in a salt solution and then taking out the sample, and the photograph of the whole Joule-heating setup is shown in Fig. S1. Finally, after water/alcohol washing several times and drying in a vacuum oven, the NiCo LDH@CC was successfully prepared, and the mass loading of NiCo LDH@CC is about ~ 1.0 mg cm^−1^. Other Ni(OH)_2_@CC, Co(OH)_2_@CC, FeOOH@CC, NiMn LDH@CC, NiFe LDH@CC and NiCoFe LDH@CC composites were synthesized through similar conditions (the experiment details at Supporting information).

### Material Characterization

X-ray diffraction (XRD) was performed on a diffractometer (D/Max-2400, Rigaku) advance instrument using CuKα radiation (k = 1.5418 Å). The microstructure and element distribution mapping of the material were characterized by scanning electron microscope (SEM, JSM-7001F) and transmission electron microscopy (TEM, JEM2100F). Raman spectra were measured on a Horiva (LabRam HR-800) spectrometer (at 532 nm, 50-mW excitation laser). X-ray photoelectron spectroscopy (XPS) was performed on a scientific ESCALAB 250 instrument. The temperature detection was tested by thermal imager (FLUKE TIS75 +) and infrared temperature instrument. The pH value of the solution was measured by a pH meter (PHSJ-6L). The content of metal elements in the sample was tested by ICP-OES (Shimadzu ICPE-9800).

### Electrochemical Characterization

Briefly, the coin cell (CR2032-type) consisted of Zn foil as the anode electrode, NiCo LDH@CC as the cathode, and 1.0 M KOH as the electrolyte. The fabrication process of Zn micro-battery can be found in the supporting information. All electrochemical tests were performed at room temperature. The energy density E (Wh Kg^−1^) and power density P (W Kg^−1^) of the battery can be obtained from the previous work formula [23]. The electrochemical properties of batteries were characterized by the Land cycler (Wuhan Kingnuo Electronic Co., China) and electrochemical workstation CHI 660E (Shanghai Chen Hua Instruments Co. Ltd., China).

## Results and Discussion

### Microstructure and Composition Analysis

**Fig. 1:**
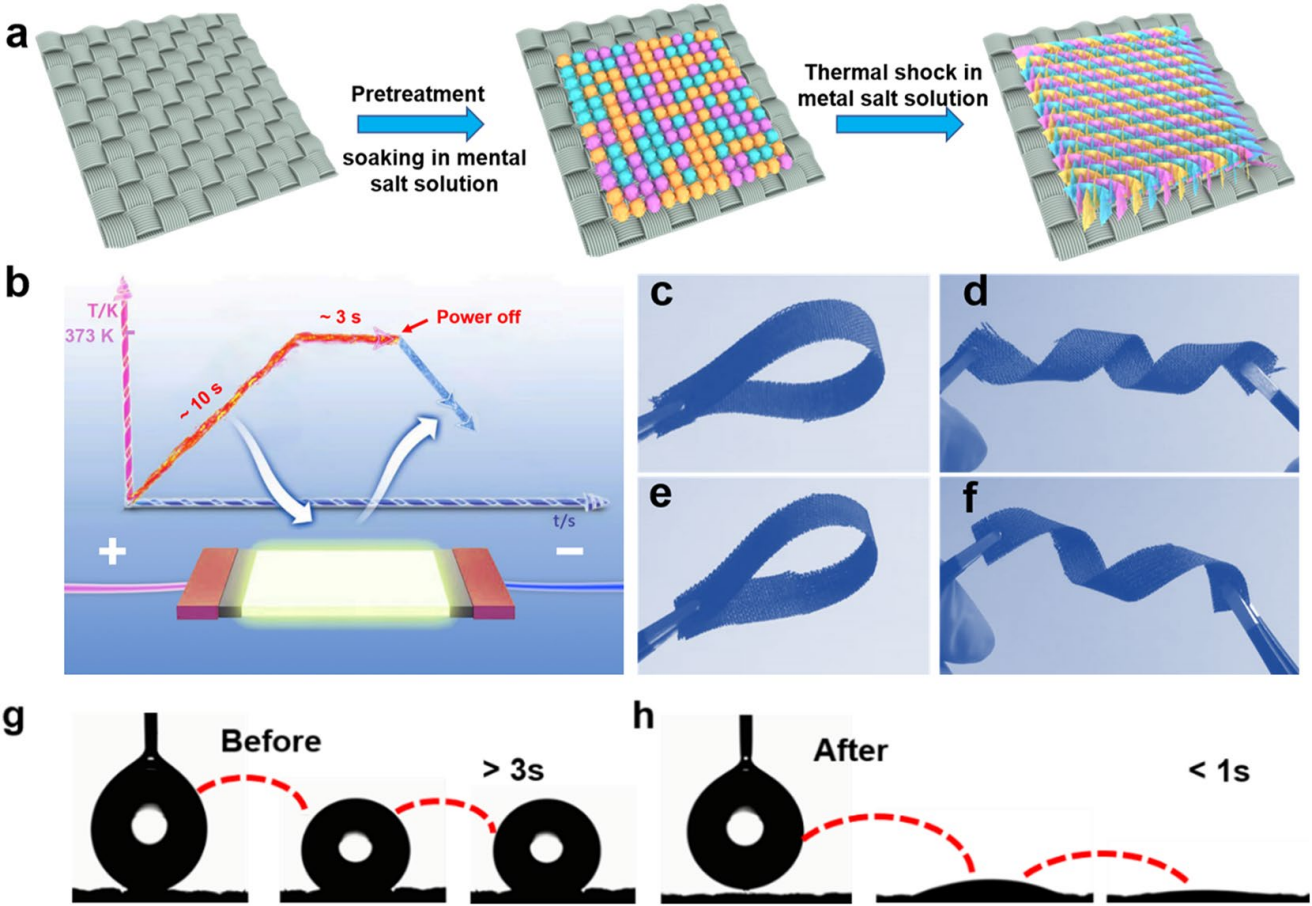
**a** Schematic illustration process of TM-LDHs@CC synthesized by using a thermal shock approach in solution system. **b** Schematic diagram of the temperature evolution of solution system with time during 13 s thermal shock. Digital images for the appearance and flexibility (bending and twisting) of **c, d** primary carbon cloth (Before) and **e, f** NiCo LDH@CC (After). Diffusion process of water molecules on **g** primary carbon cloth and **h** NiCo LDH@CC

The synthesis process of TM-LDHs on carbon cloth by using the thermal shock method is demonstrated in Fig. 1a. The temperature of the solution system can reach a high level (~ 373 K) in about ten seconds with the heating rate of ~ 8 K s^−1^ (Fig. 1b), and the 2D TM-LDHs will form and grow on carbon cloth in a few seconds under the reaction activity temperature. This preparation strategy is applicable for the synthesis of various TM-LDHs@CCs including Ni(OH)_2_@CC, Co(OH)_2_@CC, FeOOH@CC, NiCo LDH@CC and NiMn LDH@CC in an open system, and the synthesis rate can reach ~ 0.46 cm^2^ s^−1^, which is much more efficient than other methods reported in the previous works [30–32]. Such high synthesis efficiency makes it possible to realize scale-up production and even industrialization. To demonstrate the superiority of the strategy, the as-prepared NiCo LDH@CC is chosen as a typical example for further study. Interestingly, we find that the as-prepared NiCo LDH@CC perfectly inherits the good flexibility and mechanical stability of initial carbon cloth (Fig. 1c, d), which can be arbitrarily bent and twisted (Fig. 1e, f). This is also verified by the results of mechanical testing (Fig. S2). Moreover, the further hydrophilic characterization of the samples is shown in Fig. 1g, h. Compared with a static water contact angle of ∼140° after 3 s (primary CC), water droplets can be adsorbed into the NiCo LDH@CC surface within 1 s, suggesting the super wetting properties of the surfaces, which facilitates the rapid diffusion of electrolyte ions on the NiCo LDH@CC surface.

**Fig. 2:**
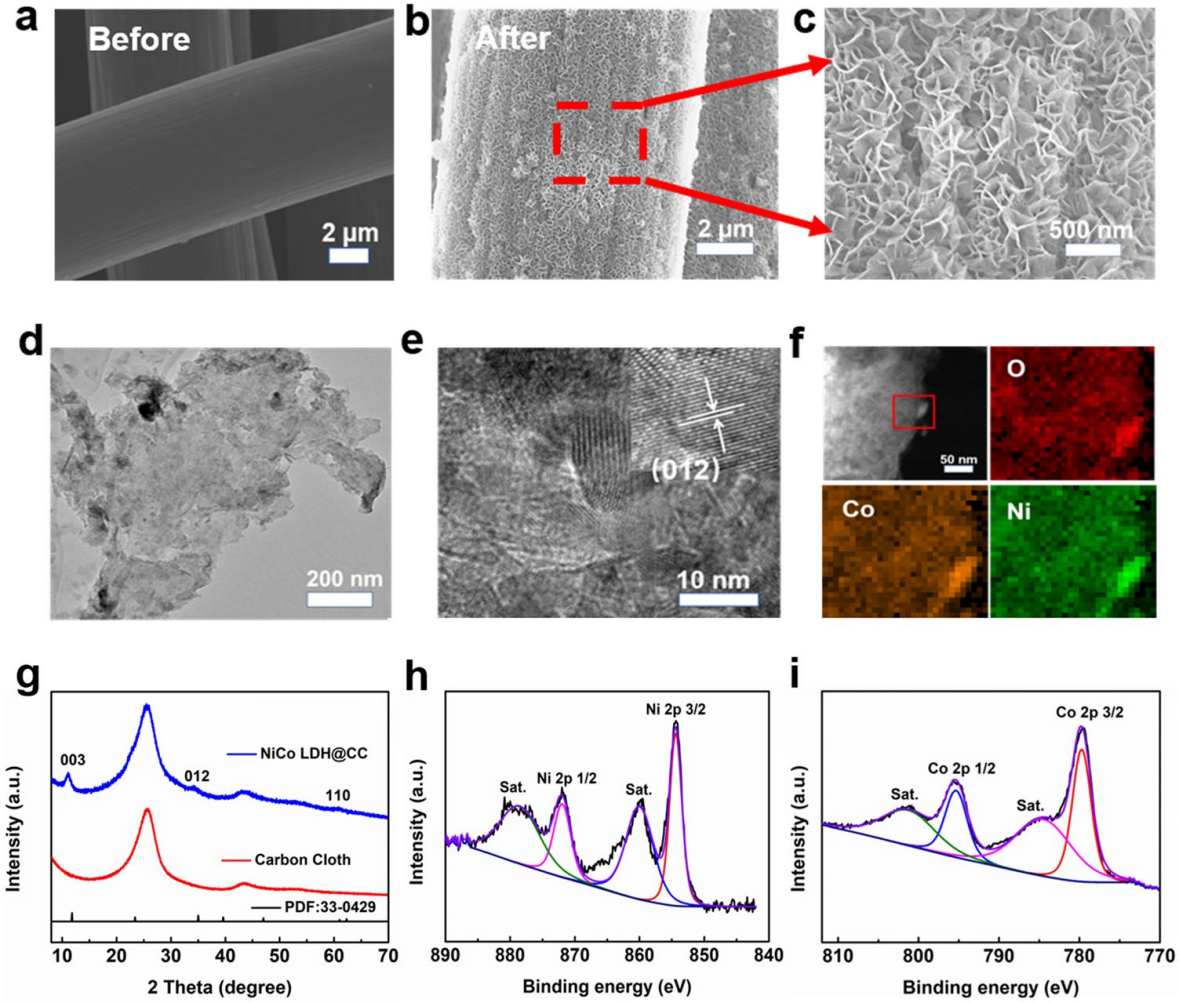
**a–c** SEM images of primary CC and NiCo LDH@CC. **d–f** TEM, element mapping and HRTEM images of NiCo LDH. **g** XRD patterns of treated carbon cloth and NiCo LDH@CC. **h–i** High-resolution XPS spectra of Ni 2*p* and Co 2*p*

To investigate the morphologies and structures of the obtained samples before and after thermal shock, the SEM and TEM characterizations were conducted. Unlike primary carbon cloth that shows a smooth and hydrophobic surface (Figs. 2a and S3), the carbon cloth treated by thermal shock for ~ 2 s exhibits a rough and hydrophilic surface (Figs. S3 and S4), greatly avoiding the sophisticated and tedious traditional pretreatment processes. Raman spectra reveal a similar value of *I*_D_/*I*_G_ that characterizes the defect degree of C atom for the untreated and treated carbon cloths (Fig. S5), indicating that the inherent conductive structure of carbon cloth is basically not damaged by instantaneous thermal shock treatment. After fast thermal shock for ~ 13 s in different precursor concentrations of solution, the microstructures of the prepared samples are shown in Figs. S6 and 2b, c. When the concentration of the NiCl_2_/CoCl_2_ precursor solution is low (e.g., 0.2 mol L^−1^), the target phase on carbon cloth exhibits rod-like nanoparticles (Fig. S6a, b). As the concentration of the precursor solution increases to 0.25 mol L^−1^, the target phase turns to a three-dimensional interconnected network by nanosheets (Fig. 2b, c), showing the promising possibility to facilitate the transport of electrolyte ions. While the concentration of the precursor solution is 0.3 mol L^−1^, the obtained samples display a granular structure formed by stacking nanosheets (Fig. S6c, d), inducing the decrease of channels for ion migration. Thus, the precursor solution of 0.25 mol L^−1^ is the optimal concentration to synthesize the target phase. The TEM image in Fig. 2d further reveals the ultrathin nanosheet structures of NiCo LDH, in which the lattice distance is 0.256 nm belonging to (012) plane of NiCo LDH (Figs. 2e and S7), suggesting the successful formation of the NiCo LDH. The uniform elemental mappings of NiCo LDH demonstrate that the nanosheets are mainly composed of Co and Ni as well as O (Fig. 2f).

The crystal structures were further confirmed by XRD patterns. As illustrated in Fig. 2g, the diffraction peaks of NiCo LDH@CC are well indexed to (003), (012), (110) planes of NiCo LDH (JCPDS No. 33–0429) compared with the treated carbon cloth [39]. Meanwhile, Raman spectra of NiCo LDH@CC (Fig. S8) also prove the existence of Ni–OH/Co–OH and Ni–O/Co–O by appearance of two detectable peaks at 464 and 529 cm^−1^, respectively [40]. XPS was applied to further investigate the composition and chemical bonding states of the NiCo LDH@CC composite (Figs. 2h-i and S9). The full survey spectrum of NiCo LDH@CC (Fig. S9a) displays the existence of Ni, Co, O, and C elements. As can be seen from the Ni 2*p* high-resolution spectrum in Fig. 2h, two main peaks at 854.9 and 873.5 eV are consistent with the binding energy of Ni 2*p*_3/2_ and Ni 2*p*_1/2_ signals of Ni^2+^, while another two peaks (indicated as “Sat”) at 862.5 and 880.6 eV indicate the existence of Ni^2+^ oxidation state on the sample. In addition, the binding energies at 781.0 and 796.80 eV belong to Co 2*p*_3/2_ and Co 2*p*_1/2_ (Fig. 2i) [41]. The O 1* s* high-resolution spectrum (Fig. S9b) illustrates two oxygen contributions, where two peaks at 531.2 and 531.7 eV can be ascribed to oxygen in OH^−^ groups coming from metal hydroxide and adsorbed OH^−^ groups at or near the surface of NiCo LDH@CC [42].

### Theoretic Prediction

**Fig. 3:**
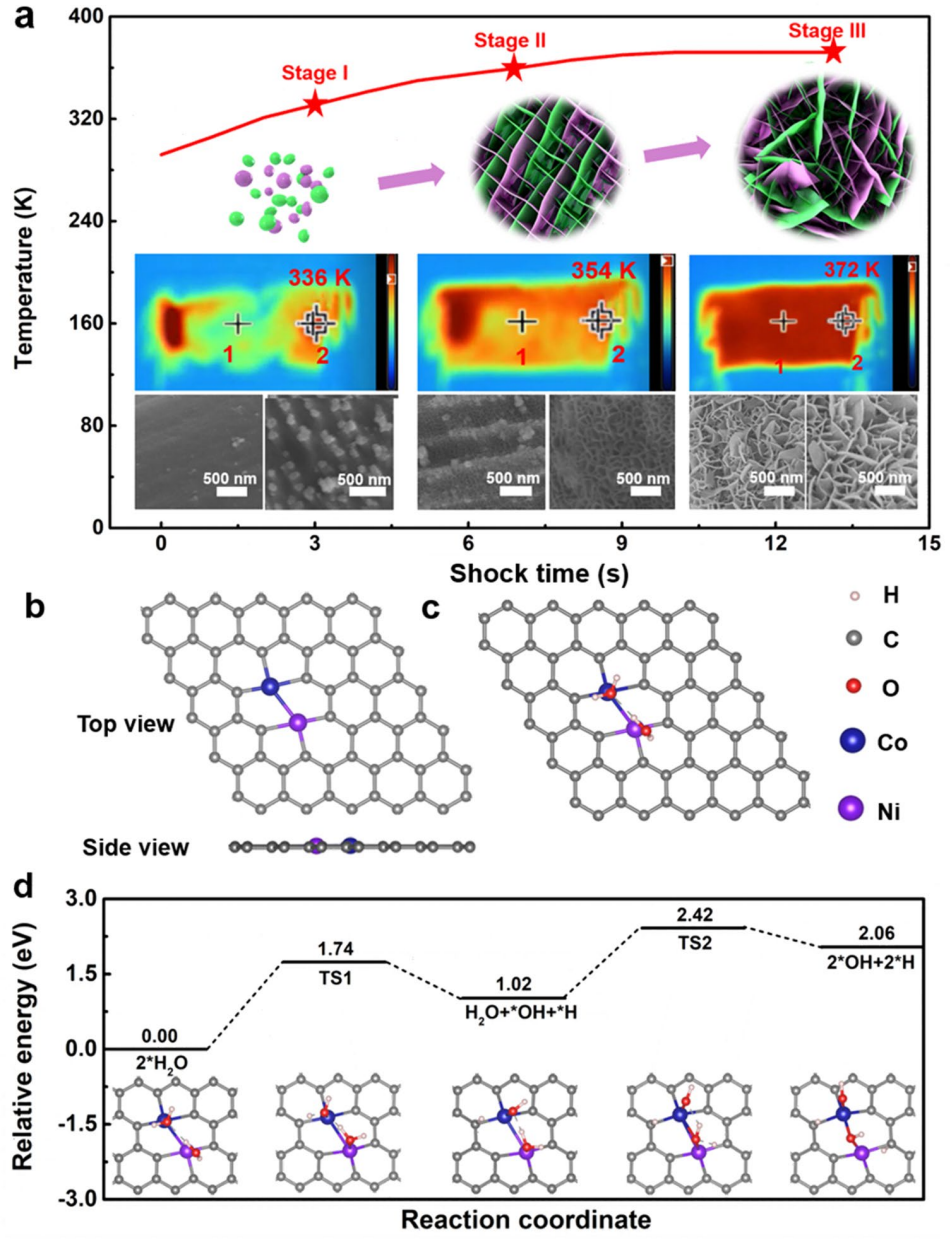
**a** Thermal images and SEM images at different markers (middle 1 and edge 2) under thermal shock (illustration 3, 7, and 13 s). **b** Structures of NiCo/graphene. **c** Adsorption structure of H_2_O on NiCo/graphene surface. **d** Energy profile for the decomposition of H_2_O, where TS1 and TS2 are the transition states

To understand the formation mechanism of NiCo LDH@CC, the growth process of NiCo LDH on carbon cloth under thermal shock (300 W) for 3, 7, and 13 s was tracked by using a thermal imager and SEM characterizations to monitor the temperature change, crystal nucleation, and growth of the whole system during thermal shock (Video S1). In the beginning of thermal shock, the temperature of whole carbon cloth substrate is around 293 K and then increases rapidly toward the center along the two ends of the carbon cloth where the positive and negative electrodes are directly connected (Fig. 3a). The SEM characterizations in the middle (region 1) and edge (region 2) areas of carbon cloth were taken to detect the microstructures of the samples during the growth process. When the time of thermal shock reaches 3 s (Fig. 3a, stage I), it is apparent that there are few nanocrystals on the surface of the carbon cloth in region 1 but a large number of nanocrystals in region 2, which may be attributed to the fast reaction kinetics as a result from higher temperature of region 2 than region 1. With the extension of thermal shock time to 7 s (Fig. 3a, stage II), it can be seen that the immature NiCo LDH nanosheets start to emerge in both region 1 and region 2. As the reaction time further increases to 13 s, the temperature of carbon cloth reaches as high as ~ 371 K, which is almost the boiling point of the solution. This nanosheet structure will further grow until the surface of the carbon cloth is uniformly covered with the NiCo LDH nanosheets (Fig. 3a stage III). With the further increase thermal shock time to 18 s, the nanosheet structure of the target phase is slightly stacked (Fig. S10a, b), but its crystallinity (Fig. S10c) is almost the same with stage III. Based on the above results, we find that the Joule heating diffuses from the edge to the middle of carbon cloth at the beginning of the thermal shock, resulting in the target phase of region 2 nucleating rapidly on the carbon cloth compared with region 1. With the progress of thermal shock, the temperature of the whole system tends to be stable, leading to the uniform deposition of the target phase on the whole carbon cloth surface. In addition, the influence of thermal effect in the growth process is investigated by conducting the thermal shock in an ice water bath. As can be seen from Video S2, the ice in the solution is almost melted completely after thermal shock for 13 s. Meanwhile, the SEM image and element mapping of the sample (Fig. S11) exhibit that the carbon cloth surface is coated with a layer of Ni-Co nanocrystals instead of nanosheets. Therefore, it can be reasonably inferred that the growth process of target phase is mainly affected by the heat effect.

Interestingly, we also find that it is hard to obtain target phase on the surface of the carbon cloth by hydrothermal method without adding additives. As shown in Fig. S12, no NiCo LDH samples can be seen on the carbon cloth after hydrothermal reaction for 8 h at 393 K, which may be due to the lack of a solution environment to generate alkaline OH^−^ ions so that the target phase is unable to be precipitated, indicating the superiority of the thermal shock strategy compared with traditional synthetic methods.

For further analyzing the mechanisms of why this fabrication process does not need to introduce additives and possesses so fast reaction kinetics. A plausible mechanism for the formation of NiCo LDH in the solution is deduced as follows:

1 $$ x{\text{Ni}}^{{{2} + }} + {\text{yCo}}^{{{2} + }} + {2}\left( {x + {\text{y}}} \right){\text{H}}_{{2}} {\text{O}} \leftrightarrow \left( {x + {\text{y}}} \right){\text{NiCo}} - {\text{LDH}} + {2}\left( {x + {\text{y}}} \right){\text{H}}^{ + } $$

where the values of *x* and y are determined by the content of metal elements. As shown in Table S1, the normalized Ni and Co elements content in NiCo LDH are approximately 26 and 74%, that is, *x* is 0.26 while *y* is 0.74. In addition, according to Eq. (1), after the thermal shock reaction, the number of hydrogen ions in the solution increases, and the pH value will decrease. Therefore, the pH values of the solution before and after thermal shock are measured by pH meter. As illustrated in Fig. S13a, the pH of the initially prepared nickel–cobalt salt solution is approximately 6.0 and decreases to 1.8 after thermal shock, which is consistent with the above equation. Additionally, it is known that the salt hydrolysis reaction is endothermic as the reverse reaction of neutralization reaction. Consequently, the temperature is crucial for the kinetics of reversible reactions. In that sense, measuring the temperature at the center of carbon cloth in the solution is helpful to explain the reaction mechanism. We measured the temperature of carbon cloth under different thermal shock power in the air medium. As shown in Fig. S13b, the power of thermal shock is linear with the temperature in the center of the carbon cloth. When the thermal shock power was 320 W, the temperature of the carbon cloth center in the solution could reach 1380 K. Based on this, we reasonably infer that the temperature at the center of the carbon cloth under thermal shock in the solution is also likely to be a very high level when the output power is 300 W. Therefore, such a high temperature from Joule heating can not only greatly enhance the reaction kinetics, but also make the reaction proceed in the direction of positive reaction, which may be the key reason for the rapid nucleation of the target phase on the substrate.

To gain insights into the formation mechanism of NiCo LDH at the molecular level, DFT calculations were performed (Details are given in the Supporting Information). The formation of NiCo LDH is modeled as the adsorption of H_2_O molecules at the Ni and Co metal centers, which are embedded in graphene, i.e., NiCo/graphene (Fig. 3b), followed with the decomposition of H_2_O that generates NiCo(OH)_2_. The binding of H_2_O molecules on NiCo/graphene is thermodynamically favored with the calculated adsorption energy of − 1.19 eV. Then, the H_2_O molecules decompose successively at Ni and Co sites (Fig. 3c). Decomposing H_2_O at Ni-site to Ni–OH and *H is 1.02 eV uphill with a barrier of 1.74 eV; further decomposing H_2_O at Co-site is 1.04 eV uphill with a barrier of 1.40 eV (Fig. 3d). Overall, the decomposition of H_2_O at NiCo/graphene is endothermic by 2.06 eV, the barrier is 2.42 eV, and the conversion energy unit is 232.68 kJ mol^−1^. According to the total load of NiCo LDH (~ 92.87 g mol^−1^) on CC (1.0 mg/1.0 cm^−2^), the energy required to break the energy barrier of reaction 1 in the whole system is approximately 15.0 J. In addition, the temperature change of the whole system from 293 to 372 K is shown in Fig. 3a, and the heating process of the whole system is about 13 s. In that way, the energy generated by thermal shock is qualitatively calculated as ~ 3,900 J, which is used in part to heat solution and in part to react. Furthermore, according to the specific heat capacity of water molecule, we can estimate that the energy needed for solution heating is approximately 3,318 J. After deducting the energy required for solution heating, the remaining ~ 582 J is much larger than the ~ 15 J needed to break the reaction barrier. Based on the above consideration, the mechanism of the whole synthesis process can be summarized as a large amount of energy obtained by thermal shock method that can break the energy of reaction 1 to drive the reaction and finally generate the target phase on the substrate. Moreover, due to the high temperature of the carbon cloth center in the solution, the reaction kinetics is greatly enhanced and the reaction time is a sharp decrease.

To explore the boundary conditions of our synthesis technology, we synthesized NiCo LDH with different powers. The SEM images of NiCo LDH@CC under different thermal shock powers from 25 to 400 W are shown in Fig. S14. It can be seen that the target structures can be formed when the power is between 70 and 300 W, but the synthesis time is slightly different. The lower the power, the longer the synthesis time. The fastest synthesis process is approximately 13 s, and the lowest synthesis process takes about more than 1 min. Theoretically speaking, when the power is greater than 75 W, the NiCo LDH@CC can be synthesized. If the power is lower than 70 W (e.g., 25 W, ~ 600 K of CC in the air, Fig. S13b), the energy generated by Joule heating would be exchanged with the environment, leading to the decrease in the temperature around reaction center. It will make the kinetics of the whole reaction very slow, and the target phase grows slowly or even hard to grow completely in a limited time (Fig. S14a). However, if the power is too high (e.g., 400 W, 1,580 K of CC in the air, Fig. S13b), the precursor containing metal-based salts will rapidly boil instantaneously along with a large number of water vapors, which may cause inhomogeneous nucleation or incomplete growth of the target phase on the CC (Fig. S14f). In addition, the XRD patterns of NiCo LDH@CC under different thermal shock power are shown in Fig. S15. When the power for preparing the samples is too low (< 130 W) or too high (> 300 W), the crystallinity of the samples is poor with no obvious characteristic peak. By contrast, the samples with good crystallinity are obtained by the preparation power of 130–300 W. Based on the above discussion, the thermal shock power between 130 and 300 W is considered a reasonable condition to fabricate NiCo LDH@CC with good crystallinity.

### Exploration of Universality

**Fig. 4:**
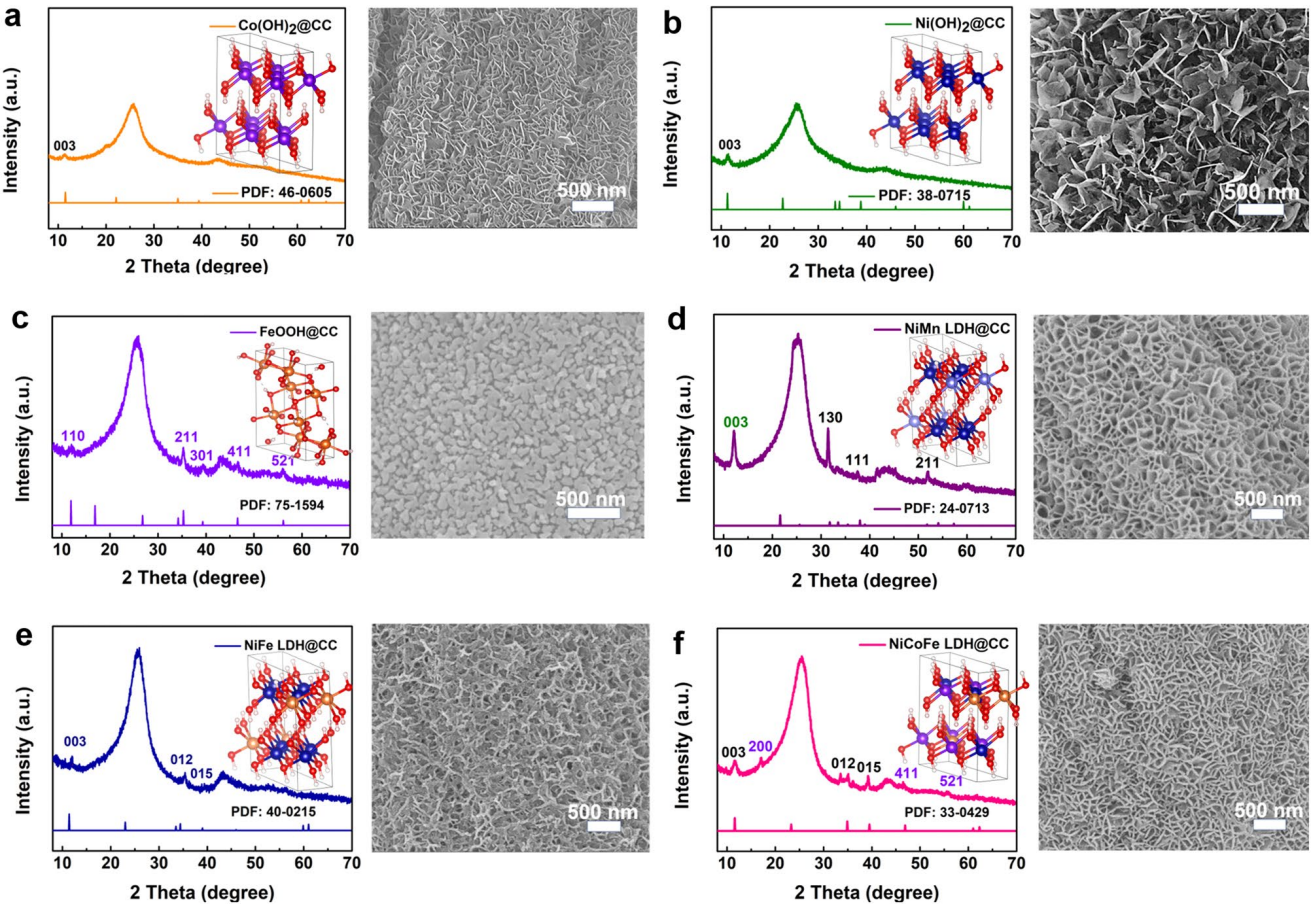
XRD patterns and SEM images of metal hydroxides based on CC **a** Co(OH)_2_@CC, **b** Ni(OH)_2_@CC, **c** FeOOH@CC, **d** NiMn LDH@CC, **e** NiFe LDH@CC, **f** NiCoFe@CC

For further investigating the generality of our synthesis technology, a series of metal hydroxides composite materials, including single transition metal LDHs (e.g., Co(OH)_2_@CC, Ni(OH)_2_@CC and FeOOH@CC), binary (NiMn LDH@CC and NiFe LDH@CC) and ternary transition metal LDH complexes (NiCoFe LDH@CC), were successfully synthesized (Fig. 4). Since the reaction mechanism of the preparation process is the hydrolysis reaction of metal salt ions, the solubility product constant of transition metal LDH complexes is a significant factor in this salt solution system. Accordingly, the single transition metal LDH without competitive deposition reaction and binary transition metal LDH complexes (NiMn LDH@CC) with similar solubility product constants (Table S2) are easy to be synthesized theoretically. The crystalline structures and morphologies of as-obtained samples are shown in Figs. 4a-d and S16a-d. The similar typical diffraction peaks at ~ 11.3° can be indexed to the LDHs phase, and the other diffraction peaks in the sample also have the corresponding attribution, demonstrating that the as-synthesized LDHs possess good crystallinity. In addition, unlike FeOOH nanoparticles, the morphologies of Co(OH)_2_, Ni(OH)_2_ and NiMn LDH present an interconnected network structure composed of nanosheets, which is consistent with previous works [27, 28, 43, 44]. It is worth noting that the solubility product constant of Fe(OH)_3_ is much smaller than that of Ni(OH)_2_/Co(OH)_2_. That is, Fe(OH)_3_ will be preferentially precipitated in the metal salt solution compared with Ni(OH)_2_/Co(OH)_2_, which is hard to get their composite phase. Thus, in our work, the sample preparation of NiFe LDH@CC and NiCoFe LDH@CC needs to be realized by stepwise thermal shock, and the specific experimental details are presented in Supporting Information. Additionally, the phase of Fe(OH)_3_ is unstable at low pH values (< 1.5, Fig. S17) and favors the formation of FeOOH with good crystallinity, which is consistent with previous reports [45]. As shown in Fig. S4e-f and S16e-f, the as-synthesized NiFe LDH@CC and NiCoFe LDH@CC possess a good crystal structure and similar interconnected network structure, further demonstrating the generality of the thermal shock process.

### Electrochemical Performance of Aqueous Zinc Ion Batteries

**Fig. 5:**
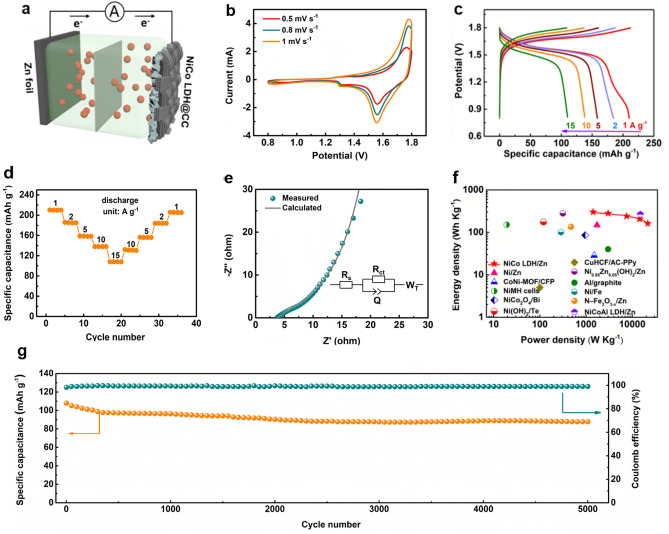
**a** Structural illustration of NiCo-LDH@CC//Zn alkaline battery. The electrochemical performances of the aqueous Zn alkaline battery. **b** Cyclic voltammetry curves at different scan rates of 0.5, 0.8, and 1.0 mV s^−1^, **c** GCD curves at different current densities of 1, 2, 5, 10, and 15 A g^−1^, **d** corresponding rate capabilities, **e** Nyquist plots, **f** Ragone plots, **g** Long-term cycling performance

Considering the ultrafast synthetic rate and high-quality crystalline phase through the thermal shock method, the NiCo LDH@CC capable of mass production is expected to be one of the promising electrode candidates for batteries in practical application. In this regard, aqueous zinc ion batteries (ZIBs) have attracted great attention due to the merits of Zn metal anode (high theoretical capacity of 800 mAh g^−1^ and low redox potential of − 0.76 V *vs* SHE) and aqueous electrolytes (intrinsic safety) [46–48]. Correspondingly, various types of systems have emerged such as Zn manganese-based batteries [49], Zn vanadium batteries [50] and alkaline Zn batteries [51]. Compared with the other two types of batteries, the rechargeable alkaline zinc-based aqueous batteries, especially the NiCo-Zn alkaline battery, are regarded as the competitive alternatives because of their high capacity, low cost and safety. Based on the above considerations, the electrochemical performances of the alkaline aqueous Zn batteries were investigated with the NiCo LDH@CC as cathode material, Zn foil as anode material, and 1.0 M KOH as electrolyte (Fig. 5a). As shown in Fig. 5b, the cyclic voltammetry (CV) curves of the Zn battery show two pairs of redox peaks without obvious shape change from 0.5 to 1.0 mV s^−1^ due to the electrochemical reaction as follow [52, 53]:

Negative electrode:

2 $$ {\text{Zn}} + {\text{4OH}}^{ - } \leftrightarrow \left[ {{\text{Zn}}\left( {{\text{OH}}} \right)_{{4}} } \right]^{{{2} - }} + {\text{2e}}^{ - } $$

Cathode electrode:

3 $$ {\text{Ni}}\left( {{\text{OH}}} \right)_{{2}} + {\text{OH}}^{ - } \leftrightarrow {\text{NiOOH}} + {\text{e}}^{ - } + {\text{H}}_{{2}} {\text{O}} $$

4 $$ {\text{Co}}\left( {{\text{OH}}} \right)_{{2}} + {\text{OH}}^{ - } \leftrightarrow {\text{CoOOH}} + {\text{e}}^{ - } + {\text{H}}_{{2}} {\text{O}} $$

Overall electrochemical reaction:

5 $$ {\text{Zn}} + {\text{CoOOH}} + {\text{NiOOH}} + {\text{2KOH}} + {\text{2H}}_{{2}} {\text{O}} \leftrightarrow {\text{K}}_{{2}} \left[ {{\text{Zn}}\left( {{\text{OH}}} \right)_{{4}} } \right] + {\text{Ni}}\left( {{\text{OH}}} \right)_{{2}} + {\text{Co}}\left( {{\text{OH}}} \right)_{{2}} $$

The change of Ni and Co valence state during charging and discharging (Fig. S18a, b) and the appearance of new peaks in *ex situ* XRD data (Fig. S18c, d) further reveal the above possible charge storage mechanism. Figure 5c displays the galvanostatic charge/discharge (GCD) curves from 1.0 to 15 A g^−1^ with the working potential window of 0.8–1.8 V. Accordingly, the specific capacity is calculated to be 211.2 mAh g^−1^ at 1.0 A g^−1^, which is larger than the counterparts of Co(OH)_2_@CC (62 mAh g^−1^, Fig. S19), Ni(OH)_2_@CC (58 mAh g^−1^, Fig. S19), other synthesis conditions (130 W and 210 W, Fig. S20) and superior to the NiCo-Zn batteries reported previously [54, 55]. The significant improvement of the electrochemical process may be attributed to the electrochemical activity optimized by the synergetic effects of Ni and Co elements [56, 57]. The rate performances of the samples at different current densities are shown in Fig. 5d. Notably, it also shows excellent rate capability under different current rates, exhibiting reversible discharge capacities of 211.2, 186, 158, 138, and 108 mAh g^−1^ at 1.0 to 15 A g^−1^ (with a capacity retention rate of 51.2%). The Nyquist plots for the device are shown in Fig. 5e, and the corresponding equivalent circuit is depicted in the inset. The electrochemical impedance spectroscopy (EIS) is consisted of a restricted semicircle (*R*_ct_ = 14.6 Ω, *R*_s_ = 3.8 Ω) and a straight line, illustrating that the device has rapid electron transfer and high ion diffusion coefficient.

To deeply evaluate the as-obtained NiCo-Zn battery, the Ragone plots are shown in Fig. 5f. Encouragingly, the device obtains a high energy of 301.7 Wh kg^−1^ at a power density of 1,423 W kg^−1^, exceeding most of the previously reported aqueous Zn batteries (Table S3) and other aqueous energy storage devices, including Ni/Zn (148 Wh kg^−1^, 1725 W kg^−1^) [58], CoNi MOF/CFP (28.5 Wh kg^−1^, 1500 W kg^−1^) [59], NiMH (151 Wh kg^−1^, 19.2 W kg^−1^) [60], NiCo_2_O_4_/Bi (85.8 Wh kg^−1^, 960 W kg^−1^) [61], Ni(OH)_2_/Te (176.3 Wh kg^−1^, 120 W kg^−1^) [62], CuHCF/AC-PPy (5 Wh kg^−1^, 100 W kg^−1^) [63] and Ni_0.95_Zn_0.05_(OH)_2_/Zn (280 Wh kg^−1^, 315 W kg^−1^) [64], Al/graphite (40 Wh kg^−1^, 3,000 W kg^−1^) [65], Ni/Fe (100.7 Wh kg^−1^, 287 W kg^−1^) [66], N-Fe_2_O_3−x_/Zn (135 Wh kg^−1^, 470 W kg^−1^) [67] and Zn/NiAlCo LDH (274 Wh kg^−1^, 16,000 W kg^−1^) [56]. Additionally, the charge–discharge cycling stabilities of the NiCo-Zn battery were also investigated at the current density of 15 A g^−1^. As illustrated in Fig. 5g, it delivers an excellent cycling performance with capacity retention of 81.4% and nearly 100% Coulombic efficiency after 5000 cycles. This is also confirmed by the no changes on structural morphologies of the NiCo LDH@CC cathode and Zn anode after long-term cycling (Fig. S21). As a proof of concept, a single NiCo-Zn battery can power a commercial electric fan (Fig. S22, Video S3).

In order to further expand the practicability of the synthetic process, graphite paper was used instead of CC as carbon substrate. As shown in Fig. S23a, b, the NiCo LDH is successfully deposited on the surface of graphite paper under the same conditions. The SEM image at high magnification displays that the wrinkled NiCo LDH nanosheets intersect to form a 3D porous interconnected network, which is similar to NiCo LDH nanosheets on CC (Fig. S23c). The elemental mapping of as-prepared samples demonstrates the homogeneous distribution of C, O, Ni and Co elements (Fig. S23d), confirming the successful fabrication of NiCo LDH@ graphite paper. Moreover, the GCD curve and EIS test (Fig. S24) of NiCo LDH@ graphite paper//Zn battery were performed to prove the practicability of NiCo LDH@ graphite paper.

**Fig. 6:**
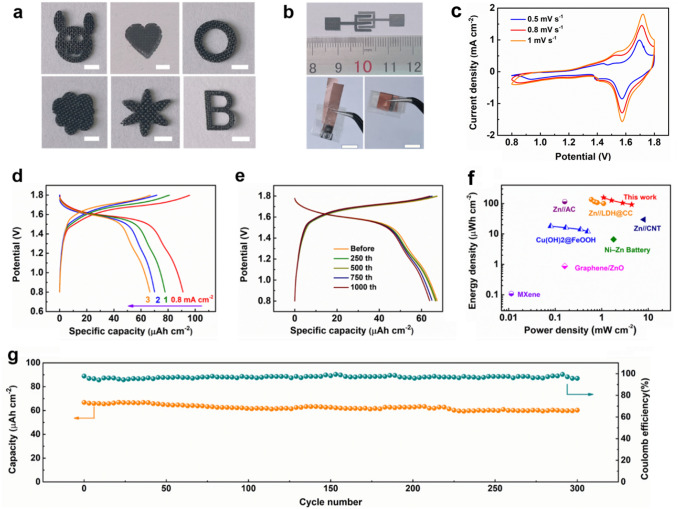
Electrochemical performance of the assembled NC-ZMBs. **a** Illustration the machinability of the as-prepared samples, scale bar: 2 mm. **b** Digital photographs of different cropped patterns from NiCo LDH@CC, a single of NC-ZMBs and a single of NC-ZMBs with bending shape and a fully crimp state (scale bar is 1 cm). **c, d** CV curves and GCD curves of NC-ZMBs at different scan rates. **e** GCD curves of the NC-ZMBs with different bending cycles under the bending angle of 180°. **f** Ragone plots. **g** Long-term cycling performance

Apart from the advantages of fast and mass production, the as-prepared NiCo LDH@CC also shows outstanding mechanical stability, flexibility, and machinability, which can be arbitrarily cut into various patterns (Fig. 6a and S25), showing potential application in next-generation emerging energy devices. For example, on-chip micropower sources (e.g., micro-batteries, micro-supercapacitors) have attracted tremendous attention in recent years due to their high energy density and great feasibility for integration into miniaturized devices, which are necessary for the development of smart medical implants, artificial intelligence robots, and other self-powered microsystems [68–70]. To this end, the interdigital electrode patterns can be customized via laser cutting. As shown in Fig. 6b, a, flexible quasi-solid-state on-chip alkaline aqueous NiCo-Zn micro-battery (NC-ZMB, the area is about ~ 0.6 cm^−2^ based on the whole device) is constructed based on interdigital NiCo LDH@CC microelectrode, Zn plate microelectrode, and PVA-KOH gel electrolyte. Notably, the NC-ZMB shows remarkable flexibility, such as bending and rotation (Fig. 6b). The electrochemical characteristic of the NC-ZMB was further evaluated by CV curves and GCD curves as shown in Fig. 6c, d. Figure 6c displays the CV curves at different scan rates from 0.5 to 1 mV s^−1^, and apparently redox signals are located at 1.68/1.58 V, suggesting reversible faradaic reactions. The small peak distance indicates low electrochemical polarization. The reaction kinetics in NC-ZMB were investigated by CV curves (Fig. S26a) according to the equation $i=a{v}^{b}$, where $i$ represents the peak current, $v$ is the scan rate and $a$, $b$ denote variable coefficients [54]. Figure S26b presents that the $b$ values calculated by the slope of the log $i$ versus log $v$ plots are 0.87 for anodic peak 1 and 0.88 for cathodic peak 2, demonstrating the dominant surface capacitive contribution in NC-ZMB. Furthermore, the capacity proportion derived from capacitive contribution is more than 73% of the total capacity, which is responsible for the high-rate capability (Fig. S26c). The GCD curves of the as-prepared NC-ZMB at different charge/discharge current densities (Fig. 6d) illustrate a broad voltage window of 0.8–1.8 V, which is consistent with the electrochemical behavior of a coin cell-type ZIB. The maximal areal specific capacity is achieved 92 µAh cm^−2^ at 0.8 mA cm^−2^. In addition, to evaluate the mechanical flexibility of microdevice, GCD measurements were taken under the condition of bending 180°. After the 1000th cycle at a 180° bend angle, the areal capacity retention of NC-ZMB is 90.8% (Fig. 6e), which manifests the remarkable flexibility of microdevice. Figure 6f depicts a Ragone plot of the assembled NC-ZMB, which obtains high areal energy of 146 µWh cm^−2^ at 1.1 mW cm^−2^, superior to the most recently reported flexible systems encompassing Zn-ion batteries and capacitors (Table S4). Figure 6g displays the cycling performance of a flexible microdevice. At 3 mA cm^−2^, a high-capacity retention rate of 91.2% is maintained after 300 cycles.

## Conclusion

In summary, a facile, scalable thermal shock method provides a theoretical guidance for ultrafast fabricating transition metal hydroxides in the aqueous phase. This may be attributed to remarkably enhanced reaction kinetics among the precursors and the rapid breaking of the nuclear barrier of the target phase during the thermal shock. Therefore, a series of well-structured TM-LDHs were successfully synthesized within dozen seconds via thermal shock. The experimental evaluations exhibited that the as-prepared NiCo LDH@CC possessed excellent electrochemical properties, including high specific capacitance of 211.2 mAh g^−1^, energy density of 301.7 Wh kg^−1^ and superior cycle stability (81.4% after 5000 cycles), when used as cathode materials of aqueous Zn ion batteries. Moreover, the further assembled flexible micro-devices have a good energy output of 146 µWh cm^−2^. Further compositional exploration (such as transition metal sulfides and phosphates) in solution system has the potential to realize broad applications.

### Supplementary Information

Below is the link to the electronic supplementary material.

Supplementary file1 (PDF 1777 KB)

Supplementary file2 (MP4 549 KB)

Supplementary file3 (MP4 469 KB)

Supplementary file4 (MP4 779 KB)
