# Supplementary material for: Ultrafast Synthesis of Metal-Layered Hydroxides in a Dozen Seconds for High-Performance Aqueous Zn (Micro-) Battery
Source: Nanomicro Lett. 2023 Jan 9;15:32. doi: 10.1007/s40820-022-01004-2 (PMC9829956; doi:10.1007/s40820-022-01004-2)
Supplement: Supplementary file 1 — Supplementary file1 (PDF 1777 KB) [file 40820_2022_1004_MOESM1_ESM.pdf]

Supporting Information for

Ultrafast Synthesis of Metal Layered Hydroxides in a Dozen Seconds for High Performance Aqueous Zn (Micro-) Battery

Xiangyang Li[1], Fangshuai Chen[1], Bo Zhao[1], Shaohua Zhang[1], Xiaoyu Zheng[1], Ying Wang[1], Xuting Jin[1], Chunlong Dai[1], Jiaqi Wang[1], Jing Xie[1,] *, Zhipan, Zhang[1] and Yang Zhao[1,] *

[1]Key Laboratory of Cluster Science Ministry of Education of China, Beijing Key Laboratory of Photoelectronic/Electrophotonic Conversion Materials. School of Chemistry and Chemical Engineering. Beijing Institute of Technology, Beijing 100081, P. R. China

*Corresponding authors. E-mail: yzhao@bit.edu.cn (Yang Zhao), jingxie@bit.edu.cn (Jing Xie)

S1 Experimental

S1.1 Synthesis of Metal LDH@Carbon Substrate Composites

$Ni(OH)_2$@CC, $Co(OH)_2$@CC and FeOOH@CC: At room temperature, 5 mM Nickel(II) Chloride Hexahydrate (Cobalt(II) chloride hexahydrate, Ferrous(III) chloride tetrahydrate) was dissolved in 10 mL deionized water under magnetic stirring. Next, the solution was transferred into a ceramic boat. A piece of treated carbon cloth was dipped into the metal salt solutions for 2 min. Then, the Joule-heating process was achieved by directly applying voltage to the both ends of treated carbon cloth for about 13 s in a salt solution. Finally, after water/alcohol washing for several times and dried in vacuum oven, the $Ni(OH)_2$@CC ($Co(OH)_2$@CC and FeOOH@CC) was successfully prepared.

NiMn LDH@CC: The synthesis method is similar to that of NiCo LDH@CC, except that is replaced Cobalt(II) chloride hexahydrate by Anhydrous manganese(II) chloride.

NiFe LDH@CC/NiCoFe LDH@CC: Since the solubility product constant of $Fe(OH)_3$ is much smaller than that of $Ni(OH)_2$, the hydroxide of iron in solution will nucleate preferentially. Therefore, We choosed two-step thermal shock to generate NiFe LDH. Briefly, we first put the carbon cloth in the solution containing Nickel(II) Chloride Hexahydrate for thermal shock to generate $Ni(OH)_2$@CC, and then washed it with water/alcohol for several times, it was placed in a salt solution containing Ferrous(III) chloride tetrahydrate for further thermal shock to prepare NiFe LDH@CC. The synthesis process of NiCoFe LDH is similar to NiFe LDH@CC.

S1.2 Preparation of the PVA-KOH Gel Electrolyte

First, 2 g Polyvinyl alcohol (PVA) was dissolved in 20 mL deionized water under stirring at 80 °C for 2 h. Then, 20 mL 2 M KOH was added slowly into the obtained

PVA solution under vigorous stirring. After cooling down, a quasi-solid-state PVA-KOH gel formed.

S1.3 Preparation of the Zn Microbattery

Briefly, as for the fabrication of positive microelectrode, the obtained NiCo LDH@CC was cut into 3 interdigital shapes with a total size of about 6 × 1 mm^2 of each finger by the computer-controlled laser cutting system. The gap between neighboring interdigitated fingers was 0.5 mm. A same method was adopted to prepare the Zn anode, except that the NiCo LDH@CC was replaced by Zn foil. And then, the planar interdigital positive and negative electrodes were transferred to the PET tape. Afterwards, PVA-KOH gel electrolyte was uniformly applied on the in-plane microelectrodes. Finally, the part of both ends of the interdigital electrode was acted as the electrode, which is bonded with the copper wire through conductive silver glue to extend the wire part, and the whole device was sealed with PET heat sealing film.

S2 Supplementary Figures and Tables

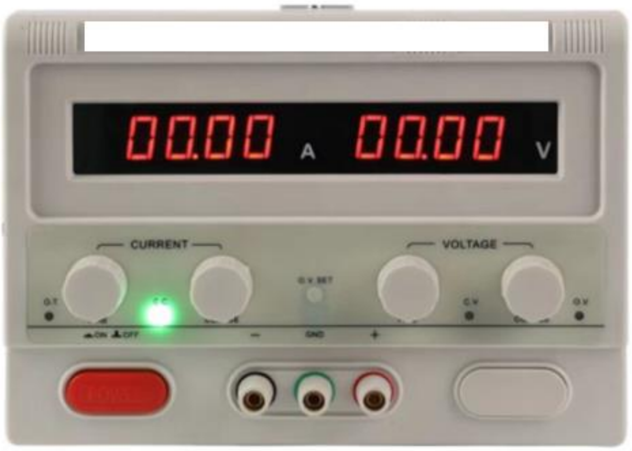

Fig. S1 Photograph of Joule heating setup

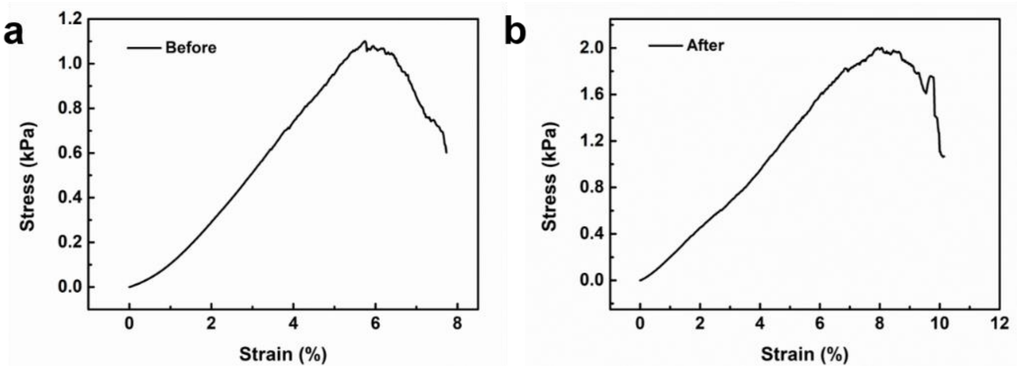

Fig. S2 The tensile stress-strain curve of **a** primary carbon cloth (before thermal shock) and **b** NiCo LDH@CC (after thermal shock)

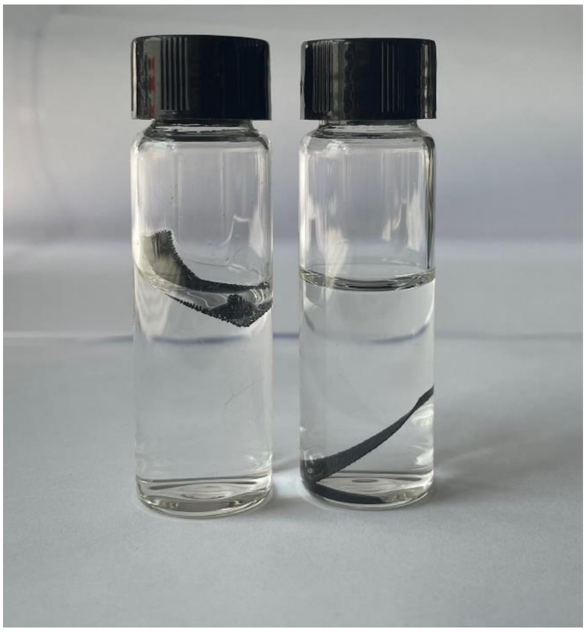

Fig. S3 Digital photo of primary carbon cloth (left) and treated carbon cloth (right) submerged in water

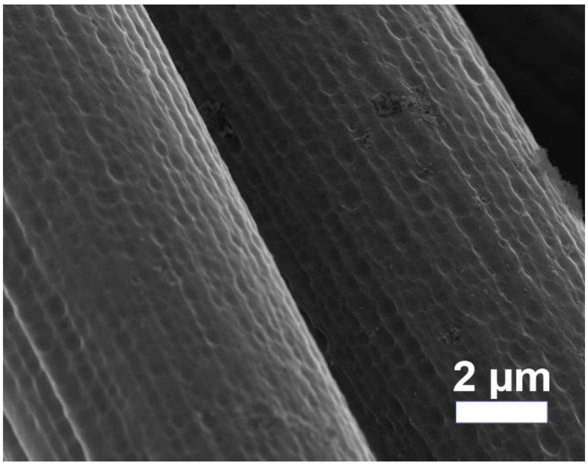

Fig. S4 SEM image of treated carbon cloth

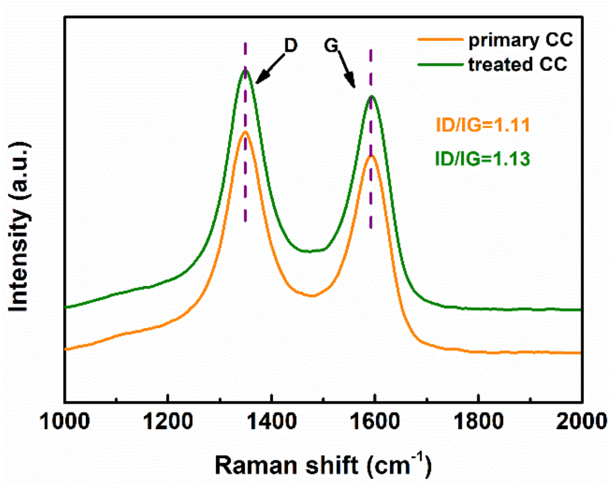

Fig. S5 Raman spectrum of primary carbon cloth and treated carbon cloth

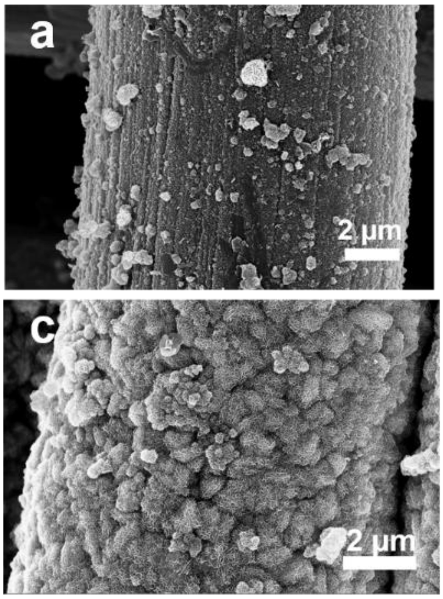

Fig. S6 SEM images of NiCo LDH@CC prepared by different precursor concentrations **a, b** 0.2 mol L^{-1} $NiCl_2/CoCl_2$, **c, d** 0.3 mol L^{-1} $NiCl_2/CoCl_2$. The yield of target phase is positively correlated with concentration of the precursor solution, approximately ~0.7 mg cm^{-2} for 0.2 mol L^{-1} $NiCl_2/CoCl_2$, ~1.0 mg cm^{-2} for 0.25 mol L^{-1} $NiCl_2/CoCl_2$, ~1.2 mg cm^{-2} for 0.3 mol L^{-1} $NiCl_2/CoCl_2$, respectively

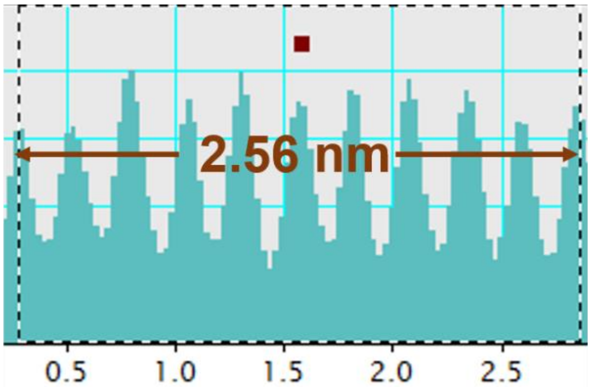

Fig. S7 Lattice space of NiCo LDH

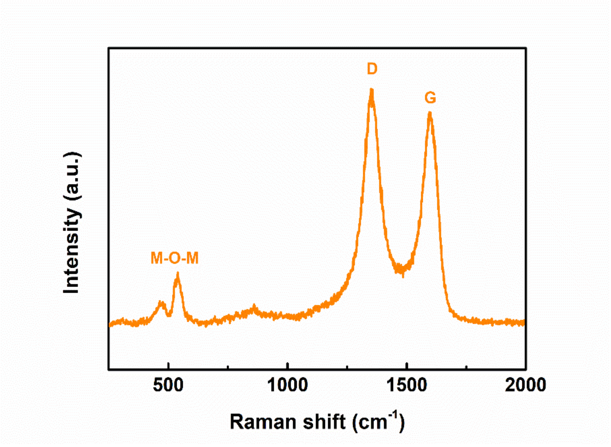

Fig. S8 Raman spectrum of the obtained NiCo LDH@CC

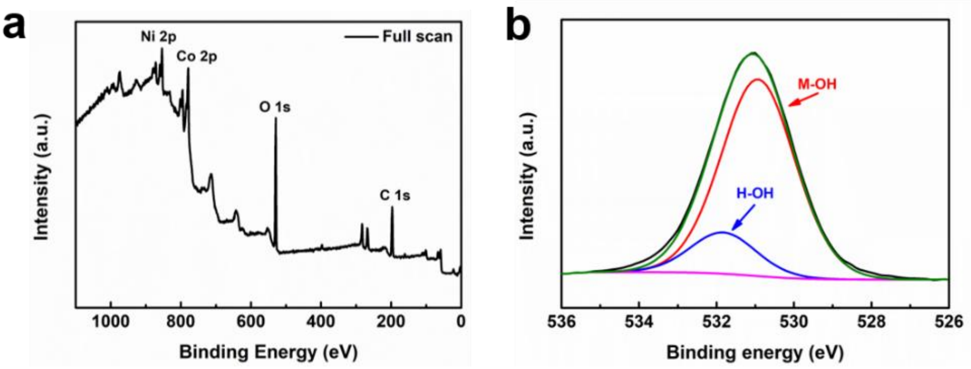

Fig. S9 XPS spectra of the NiCo-LDH@CC composite for **a** full spectra and **b** O 1s

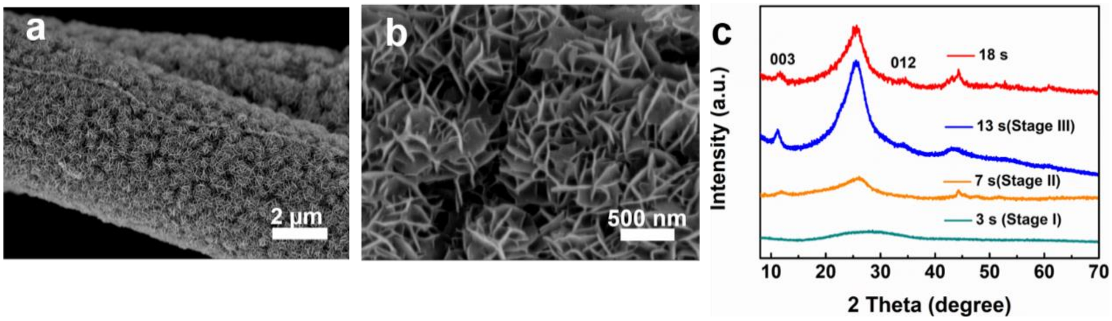

Fig. S10 a-b SEM images of the NiCo-LDH@CC (thermal shock for 18 s), **c** XRD patterns of the NiCo-LDH@CC prepared by different thermal shock time

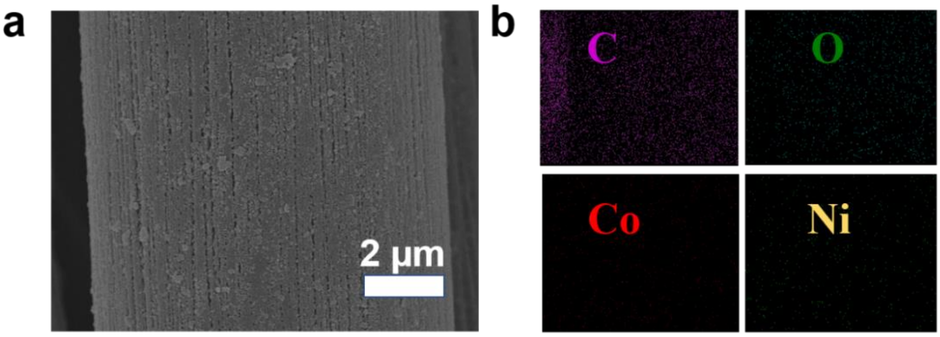

Fig. S11 SEM image and elements mapping of NiCo LDH@CC after 13 s thermal shock in nickel-cobalt solution (10 ml) with 6 g ice

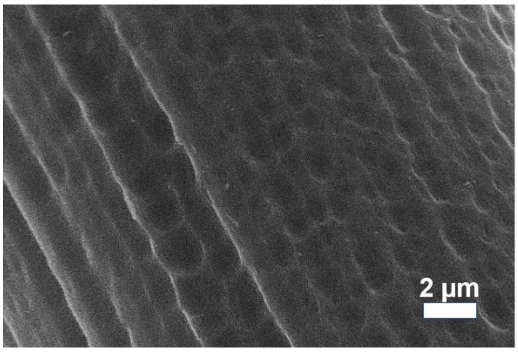

Fig. S12 SEM image of the obtained sample *via* hydrothermal synthesis method

Table S1 The molar ratio of NiCo LDH analyzed by inductively coupled plasma optical emission spectroscopy analysis (ICP-OES)

Elements	Mass contents	Normalized molar content
Co	12.67 %	74 %
i	4.37 %	26 %

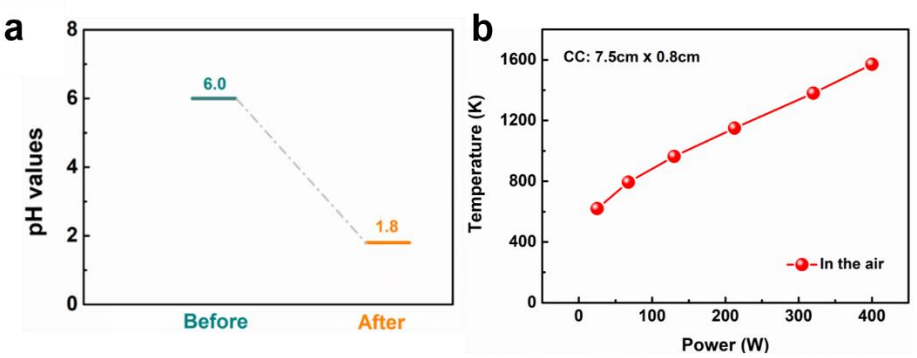

Fig. S13 a the pH vaules of the metal salt ($NiCl_2$/$CoCl_2$) solution before and after thermal shock. **b** the relationship between temperature of carbon cloth center and thermal shock power in the air

Computational methods:

Spin-polarized and periodic density functional theory (DFT) calculations are performed in Vienna Ab initio Simulation Package (VASP) software [S1, S2]. The GGA−PBE functional is used to account for the exchange-correlation interaction, with the DFT-D3 semi-empirical correction method to describe the dispersion interaction [S3, S4]. The projector augmented wave (PAW) method is utilized to describe the electrons-ionic interactions [S5, S6]. And DFT + U method is used to account for the strong correlation for the transition metals [S7], where the U parameter for Ni and Co is 3.8 eV [S8]. An energy cutoff of 520 eV and a 3 × 3 × 1 k-point mesh are used for modeled systems. Convergence criteria are set to be 10−5 eV for energy and 0.02 eV/Å for the forces. The solvation effect of water is included with an implicit solvation model by using the VASP sol tool [S9, S10], the dielectric constant of 78.5 for water is selected [S11]. The CINEB (Climbing Image Nudged Elastic Band) method [S12] is used to locate the transition state (TS) structures for H_2O decomposition, and six images are considered for the CINEB calculations. Vibrational frequency calculations are performed on the adsorbate to confirm the nature of the stationary structures. The Gibbs free energies are reported at 1300 K and computed by Eq. S1:

$$G = E_{\mathrm{DFT}} + E_{\mathrm{ZPE}} - \mathrm{TS} \qquad \text{(S1)}$$

where E_{DFT} is the electronic energy, E_{ZPE} is zero point energy, S is entropy. The latter two values are obtained by using the VASPKIT code [S13].

The adsorption free energy of H_2O is calculated by Eq. S2:

$$\Delta G_{ads} = G_{total} - (G_{surface} + G_{H2O}) \qquad (S2)$$

where G_{total}, $G_{surface}$ and G_{H2O} are the Gibbs free energies of the adsorption complex structure, the NiCo/graphene materials, and the H_2O, respectively. According to this definition, a more negative value of adsorption free energy corresponds to a stronger binding.

The decomposition free energy (ΔG_{de}) and decomposition barrier ($\Delta G^{\ddagger}_{de}$) of the H_2O are calculated by Eqs. S3-S4;

$$\Delta G_{de} = G_{*HO-H} - G_{*H2O} \qquad (S3)$$

$$\Delta G^{\ddagger}_{de} = G^{\ddagger} - G_{*H2O} \qquad (S4)$$

where G_{*H2O}, $G^{\ddagger}$, G_{*HO-H} are the Gibbs free energies of the adsorbed H_2O, transition state, and the adsorbed HO with H.

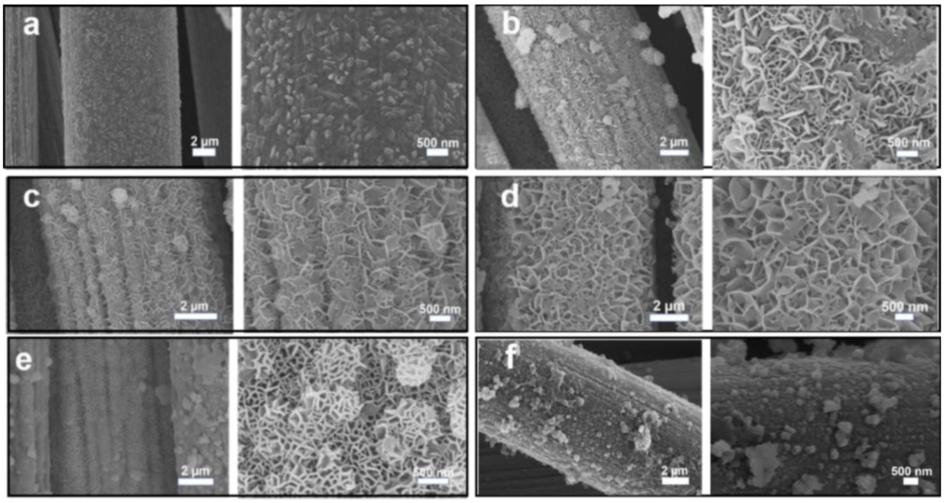

Fig. S14 SEM images of synthetic NiCo LDH@CC under different thermal shock power. **a** 25 W, **b** 70 W, **c** 130 W, **d** 210 W, **e** 300 W, **f** 400 W

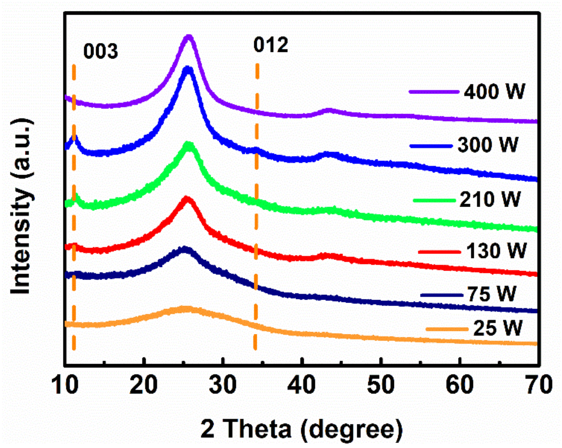

Fig. S15 XRD patterns of synthetic NiCo LDH@CC under different thermal shock power

Table S2 The solubility product of some transition metal layered hydroxides

transition metal layered hydroxides	solubility product (K_{sp})
$Co(OH)_2$	1.6×10^{-15}
$Ni(OH)_2$	2.0×10^{-15}
$Mn(OH)_2$	1.1×10^{-13}
$Fe(OH)_3$	3.2×10^{-38}

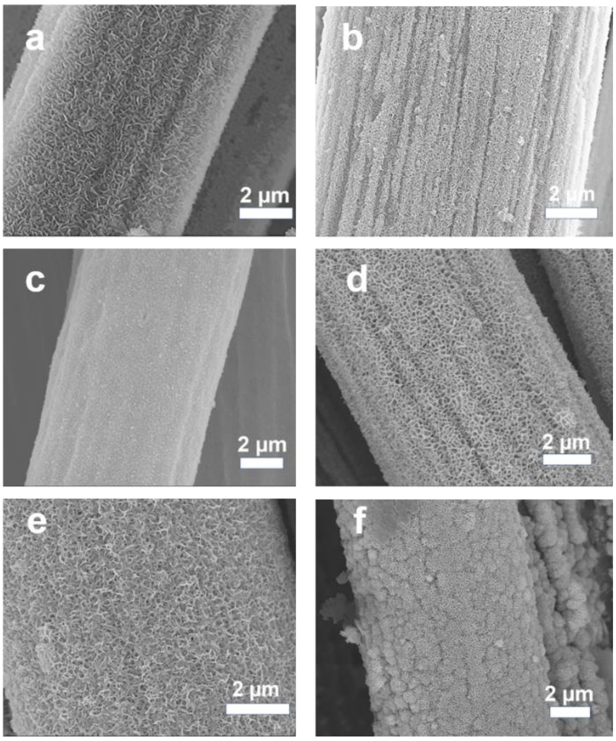

Fig. S16 SEM images of metal hydroxides based on carbon cloth **a** $Co(OH)_2$@CC, **b** $Ni(OH)_2$@CC, **c** FeOOH@CC, **d** NiMn LDH@CC, **e** NiFe LDH@CC, **f** NiCoFe@CC

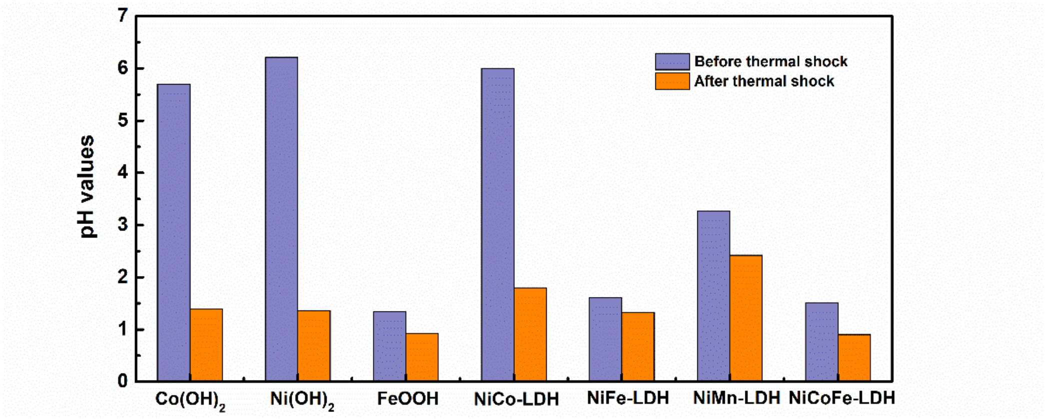

Fig. S17 pH values of the each metal salt solution before and after thermal shock

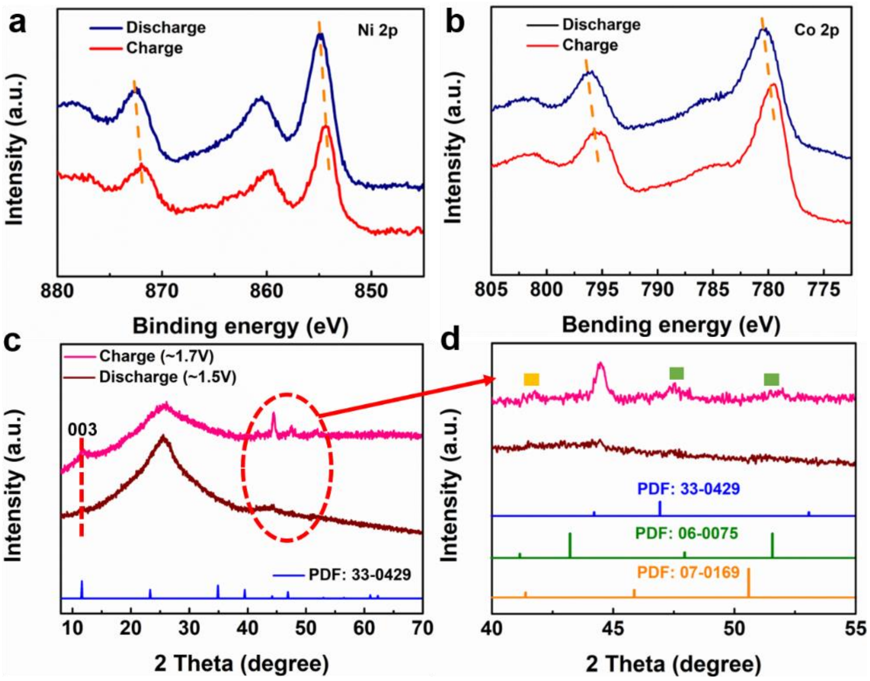

Fig. S18 a, b High-resolution XPS spectra of Ni 2p **c** and Co 2p **d** in NiCo LDH@CC at charge (+1.7 V) and discharge (+1.5 V) states

The XPS data and XRD patterns of NiCo LDH@CC during the charge/discharge processes were shown in Fig. S18. The minor shift of XPS data (Fig. S18a, b) during the charge/discharge processes further reveals the change of Ni and Co valence state (the valence increases of charging, compared with discharging), which is consistent with previous work [14]. The appearance of new peaks (Fig. S18c, d, ~48.3 ° and 51.6 ° for (107) and (108) planes of NiOOH, and ~41.1° for (006) plane of CoOOH) during charging is due to the phase transition of NiCo LDH@CC, and the weak signal of these peaks may be attributed to the strong signal of carbon peaks in CC and the low NiCo LDH loading. These results support the possible charge storage mechanism.

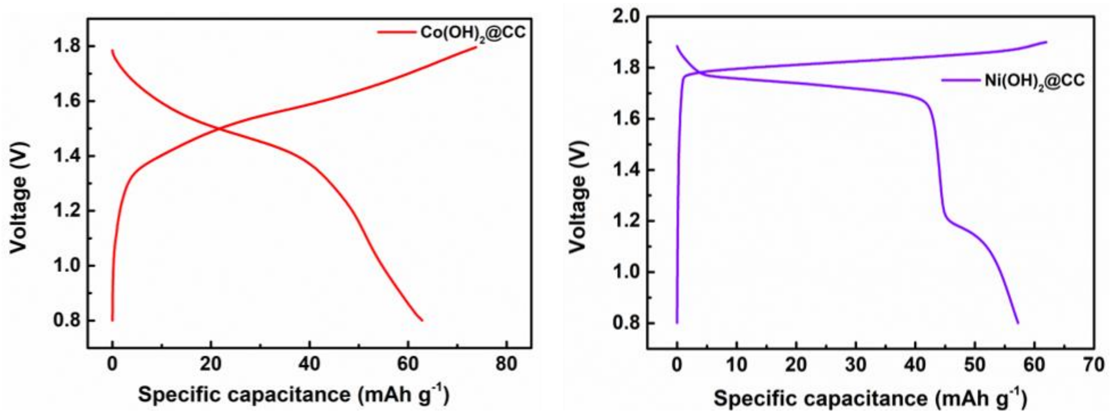

Fig. S19 GCD curves of $Co(OH)_2$@CC and $Ni(OH)_2$@CC at 1.0 A g^{-1}

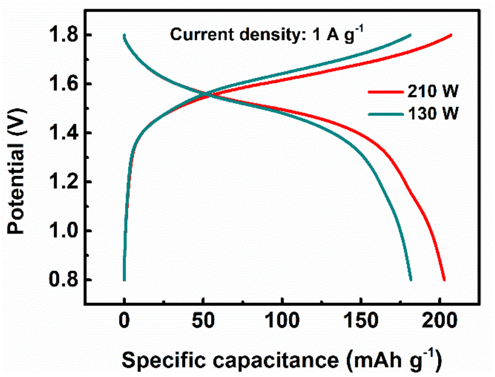

Fig. S20 GCD curves of the NiCo LDH@CC prepared by different powers (130 W and 210 W) at 1.0 A g^{-1}

Table S3 Summary the state of the art cathode materials in ZIBs and their electrochemical performance

Cathode	Specific capacity	Energy density	References
Co-doped $Ni(OH)_2$	247 mAh g^{-1} at 1 A g^{-1}	148 Wh kg^{-1}	[S15]
$Ni(OH)_2$/NiOOH	276 mAh g^{-1} at 0.29 A g^{-1}	152 Wh kg^{-1}	[S16]
$Ni_{0.95}Zn_{0.05}(OH)_2$	258 mAh g^{-1} at 0.21 A g^{-1}	275 Wh kg^{-1}	[S17]
N-Fe_2O_{3-x}	288 mAh g^{-1} at 1 A g^{-1}	135 W h kg^{-1}	[S18]
NiAlCo LDH	184 mAh g^{-1} at 3.5 A g^{-1}	274 W h kg^{-1}	[S19]
CoNi LDH	185 mAh g^{-1} at 1.2 A g^{-1}	296 W h kg^{-1}	[S20]
$Ni_3Mn_{0.7}Fe_{0.3}$-LDH	112 mAh g^{-1} at 1 A g^{-1}	–	[S21]
Ni_3S_2/NiS@NiCo-LDH	318 mAh g^{-1} at 2 A g^{-1}	556 W h kg^{-1}	[S22]
NiCo LDH	108 mAh g^{-1} at 0.49 A g^{-1}	172 W h kg^{-1}	[S23]
Co_3O_4	160 mAh g^{-1} at 1 A g^{-1}	241 W h kg^{-1}	[S24]
NiCo LDH	211 mAh g^{-1} at 1 A g^{-1}	302 W h kg^{-1}	This work

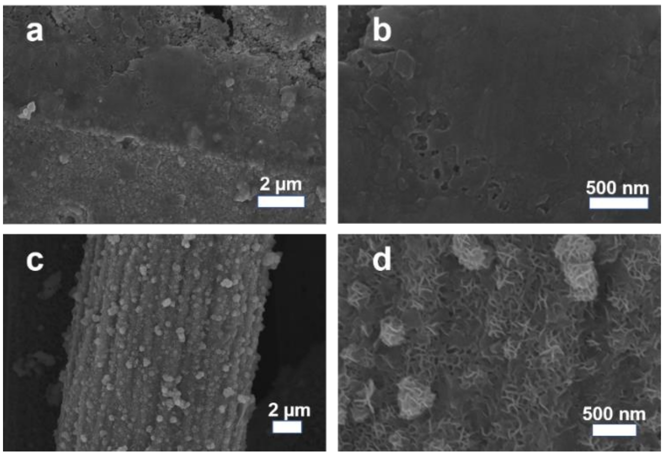

Fig. S21 SEM images of **a, b** Zn foil. **c, d** NiCo LDH@CC after 5000 cycles

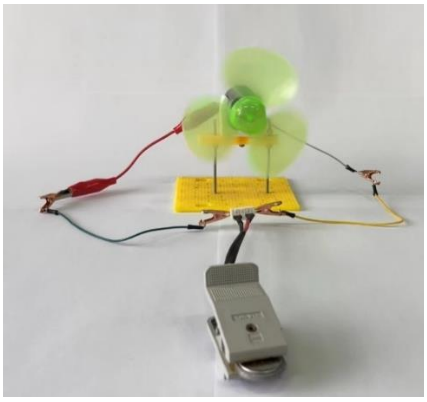

Fig. S22 Rotating optical photograph of a small electric fan powered by a single device

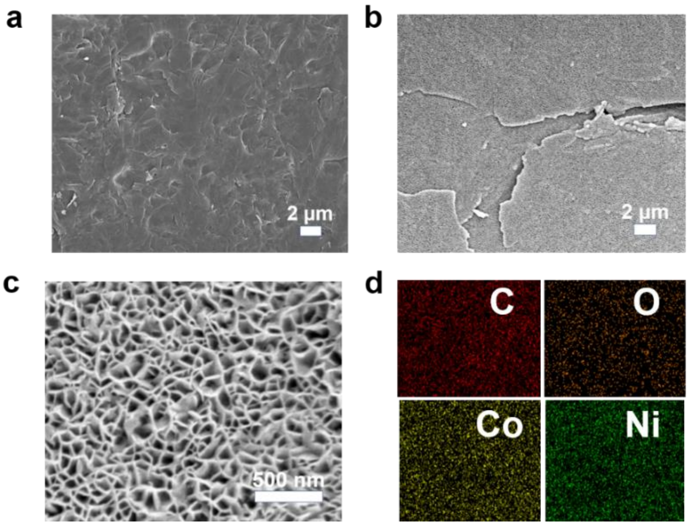

Fig. S23 SEM images of **a** graphite paper, **b, c** NiCo LDH@graphite paper, **d** the element mapping of NiCo LDH@graphite paper

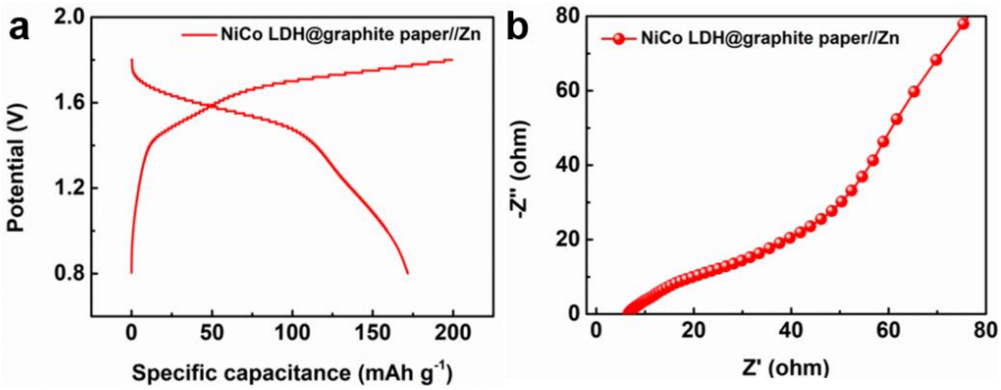

Fig. S24 a GCD curve of NiCo LDH@ graphite paper//Zn at 1.0 mA cm^{-2}, **b** Nyquist plots of NiCo LDH@ graphite paper//Zn

As shown in Figs. S24 and 5e, compared with NiCo LDH@ graphite paper//Zn, the plot for the NiCo-LDH@CC//Zn (Fig. 5e) presents a substantially smaller semicircle in the high-frequency area, indicating that it has a much smaller electron-transfer resistance due to the 3D interconnect network structure of the NiCo-LDH@CC.

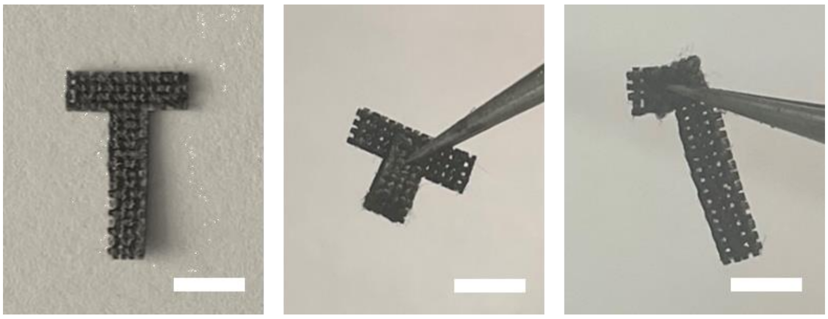

Fig. S25 Illustration the flexibility of the as-prepared samples, scale bar equals 2 mm

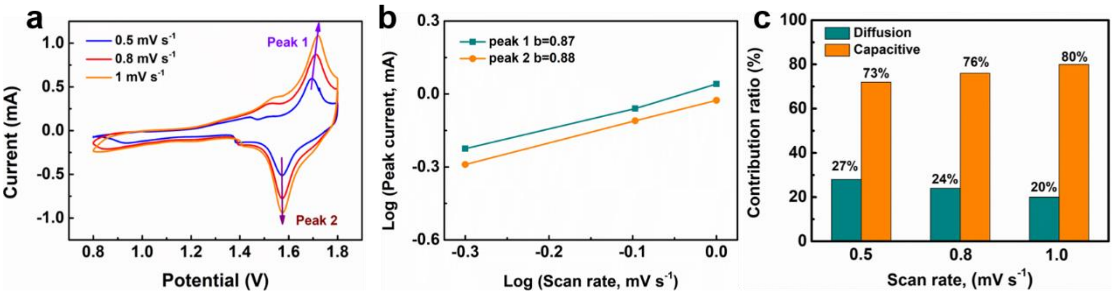

Fig. S26 Kinetics analysis of NC-ZMBs. **a** CV curves of NC-ZMBs at different scan rates of 0.5-1.0 mV s^{-1}, **b** Peak current as a function of scan rate, **c** Normalized contribution proportion of capacitive and diffusion-controlled capacity at different scan rates

Table S4 Performance comparison of recently reported in-plane energy storage devices

Cathode//Anode	Electrolyte	Energy density (μWh cm^{-2})	References
Ni-Co LDH//Zn	KOH	135.61	[S25]
Ni@Ni$(OH)_2$//Zn	6.0 M KOH+ ZnO	260	[S26]
$Co(OH)_2$@NiCo LDH// Zn	3.0 M KOH+ ZnO	170	[S27]
AC//Zn	$ZnSO_4$	115.40	[S28]
NiCoP//ZIF-C	KOH	13.90	[S29]
$Cu(OH)_2$@FeOOH	[EMIM][BF_4]	18.07	[S30]
CNT//Zn	1.0 M $ZnSO_4$	29.6	[S31]
NiCo LDH//Zn	6.0 M KOH+ 0.2 M zinc acetate	120	[S32]
NiCo LDH//Zn	1.0 M KOH	146	This work

Supplementary References

[S1] G. Kresse, J. Hafner, Ab initio molecular dynamics for open-shell transition metals. Phys. Rev. B **48**, 13115-13118 (1993). https://doi.org/10.1103/PhysRevB.48.13115

[S2] G. Kresse, J. Furthmüller, Efficient iterative schemes for ab initio total-energy calculations using a plane-wave basis set. Phys. Rev. B **54**, 11169-11186 (1996). https://doi.org/10.1103/PhysRevB.54.11169

[S3] S. Grimme, J. Antony, S. Ehrlich, H. Krieg, A consistent and accurate ab initio parametrization of density functional dispersion correction (DFT-D) for the 94 elements H-Pu. J. Chem. Phys. **132**, 154104 (2010). https://doi.org/10.1063/1.3382344

[S4] S. Grimme, S. Ehrlich, L. Goerigk, Effect of the damping function in dispersion corrected density functional theory. J. Comput. Chem. **32**, 1456-65 (2011). https://doi.org/10.1002/jcc.21759

[S5] Kresse, D. Joubert, From ultrasoft pseudopotentials to the projector augmented-wave method. Phys. Rev. B **59**, 1758-1775 (1999). https://doi.org/10.1103/PhysRevB.59.1758

[S6] P.E. Blöchl, Projector augmented-wave method. Phys. Rev. B **50**, 17953-17979 (1994). https://doi.org/10.1103/PhysRevB.50.17953

[S7] L. Wang, T. Maxisch, G. Ceder, Oxidation energies of transition metal oxides within the GGA+U framework. Phys. Rev. B **73**, 1-6 (2006). https://doi.org/10.1103/PhysRevB.73.195107

[S8] H. X. Xu, D. J. Cheng, D. P. Cao et al., A universal principle for a rational design of single-atom electrocatalysts. Nat. Catal. **1**, 339-348 (2018). https://doi.org/10.1038/s41929-018-0063-z

[S9] K. Mathew, R. Sundararaman, K. Letchworth-Weaver, T. A. Arias et al., Implicit solvation model for density-functional study of nanocrystal surfaces and reaction pathways. J. Chem. Phys. **140**, 084106 (2014). https://doi.org/10.1063/1.4865107

[S10] V. S. C. Kolluru, S. Mula, S. N. Steinmann, R. G. Hennig, Implicit self-consistent electrolyte model in plane-wave density-functional theory. J. Chem. Phys. **151**, 234101 (2019). https://doi.org/10.1063/1.5132354

[S11] A Klamt and G. Sch¨u¨urmann, 2,2-Difluoro enol silyl ethers: convenient preparation and application to the synthesis of a novel fluorinated brassinosteroid. J. Chem. Soc., Perkin Trans. J. **2**, 799-805 (1993). https://doi.org/10.1039/P29930000799

[S12] G. Henkelman, B.P. Uberuaga, H. Jónsson, A climbing image nudged elastic band method for finding saddle points and minimum energy paths. J. Chem. Phys. **113**, 9901 (2000). https://doi.org/10.1063/1.1329672

[S13] V. Wang, N. Xu, J. C. Liu, G. Tang et al., VASPKIT: A user-friendly interface facilitating high-throughput computing and analysis using VASP code. Comput. Phys. Commun. **190**, 08269 (2019).

[S14] Y. Wang, T. Wang, J. Lei and K.-J. Chen, Optimization of metal-organic framework derived transition metal hydroxide hierarchical arrays for high performance hybrid supercapacitors and alkaline Zn-ion batteries. Inorg. Chem. Front. **8**, 3325-3335 (2021).https://doi.org/10.1039/D1QI00191D

[S15] C. Xu, J. Liao, C. Yang, R. Wang et al., An ultrafast, high capacity and superior longevity Ni/Zn battery constructed on nickel nanowire array film. Nano Energy **30**, 900-908 (2016). https://doi.org/10.1016/j.nanoen.2016.07.035

[S16] Z. Ren, J. Yu, Y. Li and C. Zhi, Tunable Free-Standing Ultrathin Porous Nickel Film for High Performance Flexible Nickel-Metal Hydride Batteries. Adv. Energy Mater. **8**, 1702467 (2018). https://doi.org/10.1002/aenm.201702467

[S17] W. Zhou, D. Zhu, J. He, J. Li et al., A scalable top-down strategy toward practical metrics of Ni-Zn aqueous batteries with total energy densities of 165 W h kg^{-1} and 506 W h L^{-1}. Energy. Environ. Sci. **13**, 4157-4167 (2020). https://doi.org/10.1039/D0EE01221A

[S18] C. Teng, C. Zhang, K. Yin, M. Zhao et al., A new high-performance rechargeable alkaline Zn battery based on mesoporous nitrogen-doped oxygen-deficient hematite. Sci. China. Mater. **65**, 920-928 (2022). https://doi.org/10.1007/s40843-021-1830-2

[S19] M. Gong, Y. Li, H. Zhang, B. Zhang et al., Ultrafast high-capacity NiZn battery with NiAlCo-layered double hydroxide. Energy. Environ. Sci. **7**, 2025-2032 (2014). https://doi.org/10.1039/c4ee00317a

[S20] J. Meng, Y. Song, Z. Qin, Z. Wang et al., Cobalt-Nickel Double Hydroxide toward Mild Aqueous Zinc-Ion Batteries. Adv. Funct. Mater. **32**, 2204026 (2022). https://doi.org/10.1002/adfm.202204026

[S21] Y. Zhao, P. Zhang, J. Liang, X. Xia et al., Unlocking Layered Double Hydroxide as a High-Performance Cathode Material for Aqueous Zinc-Ion Batteries. Adv. Mater. **34**, 2204320 (2022). https://doi.org/10.1002/adma.202204320

[S22] K. Zhou, S. Wang, G. Zhong, J. Chen et al., Hierarchical heterostructure engineering of layered double hydroxides on nickel sulfides heteronanowire arrays as efficient cathode for alkaline aqueous zinc batteries. Small **18**, 2202799 (2022). https://doi.org/10.1002/smll.202202799

[S23] X. Wang, F. Wang, L. Wang, M. Li et al., An aqueous rechargeable Zn//Co_3O_4 battery with high energy density and good cycling behavior. Adv. Mater. **28**, 4904-4911 (2016). https://doi.org/10.1002/adma.201505370

[S24] H. Wang, J. Liu, J. Wang, M. Hu et al., Concentrated hydrogel electrolyte-enabled aqueous rechargeable NiCo//Zn battery working from -20 to 50 °C. ACS. Appl. Mater. Inter. **11**, 49-55 (2019). https://doi.org/10.1021/acsami.8b18003

[S25] Z. Tian, Z. Sun, Y. Shao, L. Gao et al., Ultrafast rechargeable Zn micro-batteries endowing a wearable solar charging system with high overall efficiency. Energy. Environ. Sci. **14**, 1602-1611 (2021). https://doi.org/10.1039/D0EE03623D

[S26] Z. Hao, L. Xu, Q. Liu, W. Yang et al., On-Chip Ni-Zn Microbattery Based on Hierarchical Ordered Porous Ni@Ni(OH)2 Microelectrode with Ultrafast Ion and Electron Transport Kinetics. Adv. Funct. Mater. **29**, 1808470 (2019). https://doi.org/10.1002/adfm.201808470

[S27] Y. Wang, X. Hong, Y. Guo, Y. Zhao et al., Wearable Textile-Based Co−Zn Alkaline Microbattery with High Energy Density and Excellent Reliability. Small **16**, 2000293 (2020). https://doi.org/10.1002/smll.202000293

[S28] P. Zhang, Y. Li, G. Wang, F. Wang et al., Zn-Ion Hybrid Micro-Supercapacitors with Ultrahigh Areal Energy Density and Long-Term Durability. Adv. Mater. **31**, 1806005 (2019). https://doi.org/10.1002/adma.201806005

[S29] M. Qiu, P. Sun, G. Cui, Y. Tong et al., A Flexible Microsupercapacitor with Integral Photocatalytic Fuel Cell for Self-Charging. ACS Nano **13**, 8246-8255 (2019). https://doi.org/10.1021/acsnano.9b03603

[S30] J.-Q. Xie, Y.-Q. Ji, J.-H. Kang, J.-L. Sheng et al., In situ growth of $Cu(OH)_2$@FeOOH nanotube arrays on catalytically deposited Cu current collector patterns for high-performance flexible in-plane micro-sized energy storage devices. Energy. Environ. Sci. **12**, 194-205 (2019). https://doi.org/10.1039/C8EE01979G

[S31] G. Sun, H. Yang, G. Zhang, J. Gao et al., A capacity recoverable zinc-ion micro-supercapacitor. Energy. Environ. Sci. **11**, 3367-3374 (2018). https://doi.org/10.1039/C8EE02567C

[S32] Y. Huang, W. S. Ip, Y. Y. Lau, J. Sun et al., Weavable, Conductive yarn-based NiCo//Zn textile battery with high energy density and rate capability. ACS Nano **11**, 8953-8961 (2017). https://doi.org/10.1021/acsnano.7b03322
